# Landscape genomics reveal signatures of local adaptation in barley (*Hordeum vulgare* L.)

**DOI:** 10.3389/fpls.2015.00813

**Published:** 2015-10-02

**Authors:** Tiegist D. Abebe, Ali A. Naz, Jens Léon

**Affiliations:** Department of Crop Genetics and Biotechnology, Institute of Crop Science and Resource Conservation, Rhenish Friedrich-Wilhelm University of BonnBonn, Germany

**Keywords:** landscape genomics, local adaptation, *Hordeum vulgare*, genotyping by sequencing, spatial genetic structure, adaptive loci

## Abstract

Land plants are sessile organisms that cannot escape the adverse climatic conditions of a given environment. Hence, adaptation is one of the solutions to surviving in a challenging environment. This study was aimed at detecting adaptive loci in barley landraces that are affected by selection. To that end, a diverse population of barley landraces was analyzed using the genotyping by sequencing approach. Climatic data for altitude, rainfall and temperature were collected from 61 weather sites near the origin of selected landraces across Ethiopia. Population structure analysis revealed three groups whereas spatial analysis accounted significant similarities at shorter geographic distances (< 40 Km) among barley landraces. Partitioning the variance between climate variables and geographic distances indicated that climate variables accounted for most of the explainable genetic variation. Markers by climatic variables association analysis resulted in altogether 18 and 62 putative adaptive loci using Bayenv and latent factor mixed model (LFMM), respectively. Subsequent analysis of the associated SNPs revealed putative candidate genes for plant adaptation. This study highlights the presence of putative adaptive loci among barley landraces representing original gene pool of the farming communities.

## Introduction

Natural selection is the key evolutionary process that generates the adaptation of plants to their environments (Andrews, 2010). During this, the best fitted alleles to the specific environment become prevalent through positive selection, which is the major driving force behind adaptive evolution in plants (Schaffner and Sabeti, 2008; Bose and Bartholomew, 2013). Genetic identification of those beneficial alleles is essential for answering fundamental questions concerning plant adaptive evolution as well as to utilize them in crop improvement.

Genome-wide scan has been proven to be an effective approach for studying adaptive genetic variation (Nosil et al., 2009). Classically, this approach uses different genotyping protocols to assay a large number of DNA marker polymorphisms across the genome to associate them with different traits and environmental factors (Bonin et al., 2006; Eckert et al., 2010a,b; Brachi et al., 2011; Wang et al., 2012; Westengen et al., 2012). Recently, advances in next generation sequencing technologies have resulted in the development of newer methods of high-throughput genotyping such as genotyping by sequencing (GBS). This method brought out clear advantages to genotype highly diversified and complex genomes in lesser time and at a low cost per sample (Elshire et al., 2011). GBS generates thousands of sequence tags and single nucleotide polymorphisms (SNPs) across the genome. It has been used successfully in a number of plant species like barley, maize (Elshire et al., 2011; Poland et al., 2012; Larsson et al., 2013), sorghum (Morris et al., 2013), soybean (Sonah et al., 2013), and Brachypodium (Dell'Acqua et al., 2014).

Genome-wide scan generally rely on the assumption that the loci involved in adaptation exhibit stronger differentiation among populations and lower diversity within a population when compared with selectively neutral regions of genome (Storz, 2005). Such loci are considered outlier loci and can be detected among populations using molecular marker data by calculating the population differentiation coefficient (*F*_ST_) (Excoffier et al., 2009). Therefore, *F*_ST_ analysis has the ability to determine the signatures of divergent selection evolving under the pressure of ecological factors. This selection is the fundamental process in adaptive differentiation and speciation among the natural populations of plants (Schluter, 2001, 2009; Funk et al., 2006).

Landscape genomics is a relatively new approach that combines landscape factors and genomics to scan for the presence of a signature of selection (Allendorf et al., 2010; Schoville et al., 2012). This approach attempts to detect the loci that underlie observed adaptive genetic variation and hence called adaptive loci. Currently, there is a growing body of literature demonstrating the feasibility of landscape genomics in detecting loci related to adaptation. For instance, Westengen et al. (2012) detected adaptive loci that respond to the precipitation and maximum temperature of a given habitat by analyzing African maize landrace populations using association analysis. Eckert et al. (2010b) found significant correlations between genetics and climatic variables indicating the evidence of natural selection in loblolly pine (*Pinus taeda* L.). Similarly, Poncet et al. (2010) identified ecological relevant genes linked to minimum temperatures in *Arabis alpina*. Recently, De Kort et al. (2014) reported a clear association among outlier loci, temperature and latitude in the tree species *Alnus glutinous* across Europe. These reports clearly advocate the utility of the landscape genomics in detecting and understanding the adaptive biology of plants. Dell'Acqua et al. (2014) studied local adaptation in Brachypodium and found genes related to environmental adaptation in natural populations. However, until now, the utilization of landscape genomics to dissect the fundamental components of adaptation in crops like wheat and barley has not been studied well.

Ethiopia, with its diverse agro-ecological and climatic features, is known for being one of the 12 Vavilovian centers of diversity (Vavilov, 1951; Harlan, 1969). It contains a tremendous range of altitudes spanning from 110 m below sea level in areas of the Kobar Sink to 4620 meter above sea level (m.a.s.l.) at Ras Dashen. In addition, Ethiopian regions experience huge temperature and rainfall differences, which are coupled with highly variable edaphic factors. This diverse topography and environmental heterogeneity may be the major reasons behind the highly diversified plant species across Ethiopia. These diverse climatic conditions and rich biodiversity make Ethiopia a model environment to dissect the genetic basis of ecological adaptations in plants.

Barley (*Hordeum vulgare* L.) is an important cereal for subsistence farmers in Ethiopia. These farmers typically grow barley without any application of inputs such as fertilizers, pesticides, and insecticides (Lakew et al., 1997). They usually sow their own harvested grain as seeds each year. Sowing their own seeds from year to year, these farmers have established farmer varieties (landraces) that are adapted to different ecological environments across Ethiopia. It is not possible to neglect the role of farmer-driven artificial selection to fit these landraces to a particular ecological condition. However, the prevalence and diverse adaptive differentiation of barley landraces across Ethiopia clearly suggests that these genetic resources have successfully undergone natural selection (Zeven, 1998).

The present study was aimed at detecting the signatures of local adaptation in a state of the art barley population using the landscape genomics approach. Here, we report the first insight into the identification of putative adaptive loci by combining molecular data of diverse barley landraces with highly divergent climatic variables. The detection of these signatures of local adaptation in a long-lasting native barley gene pool of the farming communities, will help in understanding the mechanisms of plant adaptation in barley and beyond in major crops like wheat.

## Materials and methods

### Plant material and genotyping

**Figure 1:**
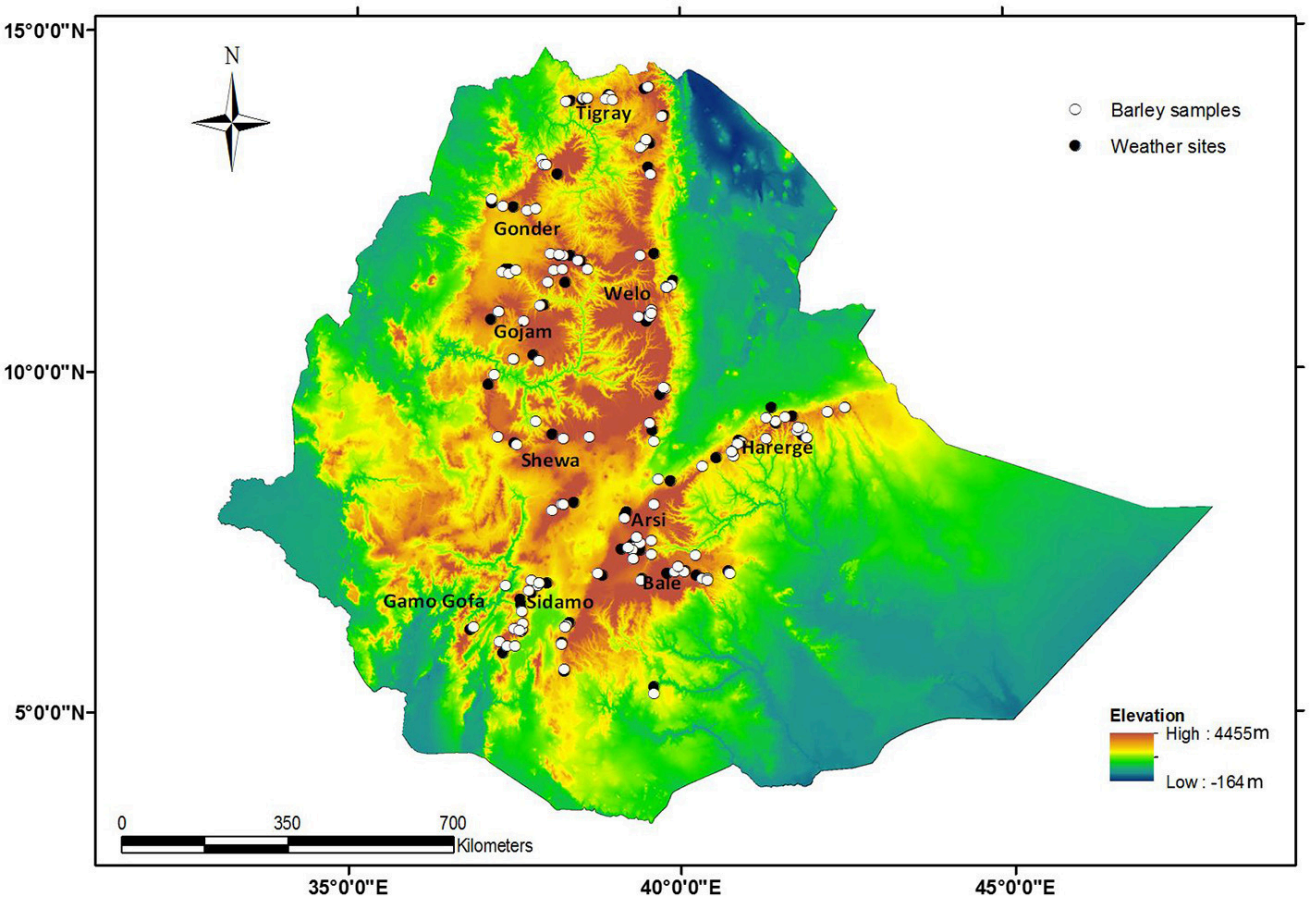
**Distribution of barley landraces and weather sites across Ethiopia on an altitudinal map**.

In the present study, we selected 130 diverse barley landraces originating from 10 major barley-growing regions of Ethiopia (Figure 1). These landraces are not only described with altitude and geographic coordinates but also with the vernacular name given by the local community. This germplasm and its detailed information were provided by the Institute of Biodiversity Conservation (IBC) in Ethiopia. We genotyped two samples from each landrace resulting in 260 total samples (Table S2), which were analyzed using the genotyping by sequencing (GBS) approach. In addition, a German spring barley cultivar Barke was included in two replications as an internal control for the GBS analysis and data control. Initially, all samples were planted in a glass house, and after 2 weeks, the leaves were harvested for DNA extraction using the Qiagen DNeasy plant mini kit (Qiagen, Hilden, Germany) to ensure high-quality DNA, which was required for the GBS analysis. After DNA extraction, GBS libraries were prepared and analyzed at the Institute for Genomic Diversity (IGD), Cornell, USA, according to Elshire et al. (2011) using the enzyme *PstI* for digestion and creating a library with 96 unique barcodes. These libraries were sequenced using the Illumina HiSeq2000 platform. GBS analysis pipeline ver. 3.0.139, an extension to the Java program TASSEL (Bradbury et al., 2007), was used to call SNPs from the sequenced GBS library with the following options. Tags were aligned with the barley reference genome of cv. Morex (International Barley Genome Sequencing Consortium, 2012). VCF tools ver. 0.1.8 (Danecek et al., 2011) was used to summarize and filter data as well as to generate input files for PLINK (Purcell et al., 2007), which were used for MDS (multidimensional scaling). The output was visualized using basic plotting functions in R ver. 2.15.0 (R Development Core Team, 2008). Before using these SNP markers for analysis, the original SNP data were filtered by applying different criteria. The first criterion was the SNP call rate for which SNP markers showing less than 10% missing values were passed to the next step. Among these, SNPs with a minor allele frequency (MAF) of less than 5% and monomorphic SNPs were excluded from the data. However, two barley samples (1%) were excluded in the final analyses because of missing genotypic data.

### Climatic data

The climate data from 61 weather sites were provided by the Ethiopian Meteorological Agency (Figure 1). The weather data were collected over multiple years, for an average of 21 years. The weather sites supplied monthly rainfall (lm^−2^) and maximum and minimum temperature (°C) data. The three main seasons of Ethiopia, *Kiremt* (June–September), *Bega* (October–January), and *Belg* (February–May) were the basis for the grouping of the annual climatic data (USDA, 2002). *Kiremt* is the main rainy season all over Ethiopia, whereas *Bega* is the dry season, and *Belg* is considered the short rainy season. The altitude data were obtained from the passport data of the barley samples procured from the Institute of Biodiversity Conservation of Ethiopia (IBC). The altitudes of the sampling sites were grouped into four classes according to the traditional agro-ecological classification of Ethiopia. These classes are cold temperate, cool sub-humid highlands (Classes I and II, 1500–2500 m.a.s.l.), cool humid highlands (Class III, 2500–3000 m.a.s.l.) and highlands (Class IV, over 3000 m.a.s.l.) (USDA, 2002). The temperate, cool sub-humid highland was further divided into two classes because it covers a wide range of altitudes (Table S1).

### Inference of population structure

Correction of the confounding effect of population structure in association studies plays a major role in reducing false positives (Pritchard et al., 2000a; Yu et al., 2006; Kang et al., 2008). Similarly, detecting adaptive loci without considering the impact of population structure will lead to false positive loci. Therefore, the analysis of hierarchical population structure was computed using the Bayesian-based program STRUCTURE ver. 2.3.5 (Pritchard et al., 2000b). For the analysis, an admixture model with correlated allele frequencies was chosen (Falush et al., 2003). The analysis was performed for a number of subpopulations varying from *K* = 2 to *K* = 20. For each value of K 20 independent runs were performed. For each run a burn-in of 10,000 and 50,000 iterations was specified. Finally, the Evanno et al. (2005) method was applied to determine the number of K. For this function, a web-based program, STRUCTURE HARVESTER ver. 0.9.93 (Earl and Vonholdt, 2012) was employed to infer the level of population structure. Ultimately, CLUMPP (Jakobsson and Rosenberg, 2007) was used to combine and average the individual's assignment across 20 runs for the determined number of K. To identify barley landraces that were admixed, each individual sample was assigned to its respective group based on a membership coefficient. The samples with a membership coefficient of ≥90% were assigned to a single group, whereas those that were smaller than the threshold were considered admixed. The membership coefficients (Q) were calculated using administrative regions instead of considering LOCPRIOR option during structure analysis. Eventually, altitude classes were used as basis of grouping to test if the detected sub-populations were influenced by altitude. This was determined by, assigning each barley accession to its origin of altitude class and plot the structure graph using the membership coefficient.

### Principal component analysis

A principal component analysis (PCA) was conducted using SNP markers data to reduce the number of variables into fewer components that explain the maximum variance. These components were then plotted in a two-dimensional plot for ease of viewing the existing genetic pattern. Before computing the PCA, the missed marker data were replaced with the mean values calculated over the markers. Subsequently, the analysis was carried out with the Proc princomp procedure using SAS software ver. 9.3 (SAS, 2011). A parallel analysis (PA) (Franklin et al., 1995) was then carried out to decide the number of principal components to retain for further analysis. PA is a method based on the generation of random eigenvalues to determine the number of components to retain. The eigenvalues are computed from the permutations of the observed data rather than from simulated data. This is an advantage not to keep the assumption of multivariate normality since the null reference set is conditioned on the observed data (Ledesma and Valero-Mora, 2007). In this analysis the covariance matrix was decomposed in which the parallel analysis restricted random matrices to have variable means and standard deviation of the real data (Franklin et al., 1995). Hence, a permutation test of 100 replications was used to run covariance matrices to calculate the eigenvalues. Afterwards, principal components which showed higher observed eigenvalues than their randomly generated associated values were retained for further analysis.

### Spatial genetic structure

Isolation by distance (IBD) was computed using the “Spatial” option implemented in GenAlEx ver. 6.41 (Peakall and Smouse, 2006). The autocorrelation coefficient (r) obtained was similar to Moran's *I* (Moran, 1950), which ranges from -1 to 1. The spatial autocorrelation analysis was computed based on the pairwise comparison of the genetic distances derived from the genetic markers and geographic distance (km). Prior to performing the correlation analysis, the coordinates were converted into the Universal Transverse Mercator (UTM) system, and autocorrelation was computed first for all accessions from all regions followed by another analysis excluding accessions collected from Tigray. Accessions collected from Tigray region were excluded because of the geographic distance of the region and the sole grouping of the accessions during structure analysis. The significance of the spatial autocorrelation value was tested by constructing a two-tailed 95% confidence interval around the null hypothesis of no spatial genetic structure, which is *r* = 0. The analysis was performed with an option of an even distance class of 20 km based on a study that reported the distances traveled by Ethiopian farmers to obtain seeds (Bishaw, 2004). Permutations of 9.999 and a bootstrap of 1000 were used to compute the confidence interval around the null hypothesis.

### Partitioning of genomic variation due to climate variables and geographic distance

Partial redundancy analysis (RDA), a constrained ordination technique, attempts to explain differences in species composition by combining a regression analysis with a principal component analysis (Borcard et al., 2011). It is based on genetic and environmental matrices (climate and geography). Partial constrained ordinations determine relationship between desired environmental and biological variables by removing the effect of known and uninteresting factors. Whereas unconstrained partial RDA considers the residual variance (Peres-Neto et al., 2006). In the present study, RDA was computed using XLSTAT ver. 2014.05.1 and *vegan* function in R package to disentangle the relative contribution of climate variables and geographic coordinates in driving genetic structure (Legendre and Fortin, 2010). For this, Hellinger transformed SNP allele frequencies were used as the response variable, and climate and coordinates as explanatory variables (Liu et al., 2011; De Kort et al., 2014). Before running the analysis the climate data were standardized using the *Proc* Stand procedure in the SAS software. The geographic variables were also normalized using a square root transformation of the geographic coordinates (Borcard et al., 2011). To examine how much of the genetic variation in barley landraces explained by climate variables, geographic coordinates and the combination of both, the variance components of the RDA were partitioned by running three different models. The first model considered all climate and geographic variables as explanatory variables (Model 1); the second model was a partial model in which the climate variables explained the genetic data conditioned on geographic coordinates (Model 2); and the third model was a partial model in which geographic coordinates explained the genetic data conditioned on climate variables (Model 3). For all models redundancy analysis was followed by significance test using Monte Carlo permutations test with 500 runs. For determination of best model forward selection with permutation of 999 and α = 0.01 were computed. This process of model determination was improved by the introduction of adjusted *R*^2^ by Peres-Neto et al. (2006), and the analysis was conducted using *ordistep* function of *vegan* in R package (Oksanen et al., 2010). Subsequently, the variation partitioning was followed when more than one significant explanatory variables were found (Legendre and Legendre, 1998).

### Association analysis of climatic variables

At present, a number of statistical tools are available for detection of outlier loci that are possibly affected by selection (Pérez-Figueroa et al., 2010; Narum and Hess, 2011). In the present study, we used two different software for the associations, between the environments and SNPs, and one for detection of the outlier loci. Bayenv2 and latent factor mixed model (LFMM) were used to identify association of climate factors with genetic markers whereas outlier loci were detected using BayeScan software. A detailed description of each statistical method is presented below.

The detection of loci correlated with different climatic variables was carried out using Bayenv2 (Coop et al., 2010) and LFMM (Frichot et al., 2013). Bayenv is a Bayesian method that estimates the empirical pattern of covariance in allele frequencies between populations from a set of markers and then uses this as a null model for testing individual SNPs. Genome scans for SNPs with allelic correlations with climate variables were performed using Bayenv2 (Coop et al., 2010; Günther and Coop, 2013). This program runs in two steps. First, it creates a covariance matrix of relatedness between populations. Then, in the second step, it runs the correlation between the covariance matrix and the environmental variables generating a Bayes factor (BF) and non-parametric Spearman's rank correlation coefficient [ρ (Rho)]. The null model assumes that allele frequencies in a population are determined by the covariance matrix of relatedness alone against the alternative model, where allele frequencies are determined by a combination of the covariance matrix and an environmental variable, producing a posterior probability (Coop et al., 2010). Before running a null model estimation, the exclusion of outlier loci and loci which are in linkage disequilibrium, is recommended to ensure independence between SNPs on a chromosome (Bayenv2 Manual). Hence, we excluded outlier loci which were detected using BayeScan and LFMM program followed by loci which were in linkage disequilibrium (*r*^2^ > 0.2) within each linkage group. The rest (801 neutral SNPs) were used to estimate the covariance matrix with 50,000 iterations. To control the variation across the covariance matrix, the average was calculated for the outputs of 10 matrices. Covariance matrices were compared after three independent runs with different seed numbers to ensure that the matrix was well-estimated. According to the recommendation of Blair et al. (2014) the BF of each SNP was calculated by averaging five independent runs of Bayenv2 at 50,000 Markov chain Monte Carlo (MCMC) for both the covariance matrix and Bayes factor analysis. For detection of outlier loci, Günther and Coop (2013) recommended considering the Spearman correlation coefficient, which measures the correlation between ranks of SNP allele frequencies and environmental factors, in addition to BF. BF is considered to have a slightly higher power, and SNPs, which fall in the top x% of BF and y% (where x < y; Bayenv2 Manual) of absolute values of spearman rank correlation coefficient ρ, are suggested to be robust candidate loci. Thus, we considered loci which were commonly detected in the top 1% of the *BF*-values (*BF* > 3) and top 5% of the absolute correlation values as a significant putative adaptive loci.

The other correlative method used for adaptive loci detection was LFMM, a software package that is a newly developed statistical model (Frichot et al., 2013). According to the study conducted by de Villemereuil et al. (2014), LFMM provided the best compromise between power and error rate across different scenarios. LFMM tests the association between environmental and genetic markers while estimating the hidden effect of population structure. The LFMM implemented fast algorithms using a hierarchical Bayesian mixed model based on a variant of PCA, in which the residual population structure is introduced via unobserved or latent factors. All SNP markers (1370) and the original climate variables were used for association analysis. The principal components of environmental variables are recommended when the summary of the variables is required because of their numbers (personal communication with Dr. Eric Frichot). The first three principal components generated for genetic markers were used as latent factors to estimate the population structure effect. The SNPs, which showed an association with environment, were determined based on the z-score. To estimate the z-scores for the environmental effect, the Gibbs sampler algorithm was run for 50,000 sweeps after a burn-in period of 10,000 sweeps. The threshold for the z-scores was determined after applying the Bonferroni correction for type I error α = 0.01. Loci exhibiting z-scores above the absolute value of four and corresponding to *P* < 10^−5^ were retained as significant loci.

### Outlier loci detection

BayeScan is the tool that we used to detect outlier loci. It is a Bayesian based method that depends on a highly differentiated locus (Foll and Gaggiotti, 2008). It is the most conservative method with the least type I error compared to other outlier loci detection methods (Narum and Hess, 2011). However, it may detect high false positive loci if demographic history is not included in the analysis (Lotterhos and Whitlock, 2014). BayeScan identifies loci that are characterized by higher or lower levels of population divergence than neutral loci, suggesting a diversifying or purifying selection. It estimates the probability that a given SNP is under selection by calculating the posterior odds (PO). The PO are the ratio of the posterior probabilities of the two models (selection/neutral) for each locus based on the allele frequency. Before running the outlier loci analysis, the barley landraces were assigned to their respective K groups, thus supporting the comparison of the discrete groups in the process of candidate loci detection. To compare the result of outlier analysis, the individuals were assigned twice based on admixture Q coefficients of ≥70 and ≥90. Outlier loci detection was conducted by setting the prior probability of the model with a selection of 1/10, assuming a priori that the neutral model is 10 times more likely than the model including selection. During this run, all of the default values of 10 pilot runs of 5000 iterations with 50,000 additional burn-in steps were retained. We used false discovery rate (FDR = 0.05) as significance level for detection of the outlier loci. The FDR was controlled using the *q*-value which is the FDR analog of the *p*-value (Storey, 2002).

### Detection of candidate genes

Candidate genes were found using the BLASTn function of DNA sequence analysis where the DNA sequences of SNP markers showing significant association were searched against the barley genome sequence using the NCBI and IPK databases. Genomic contigs showing the best hits were selected based on highly significant and maximum similarity percentages (>95%) and an *E*-value cut-off of 1E-15. The putative candidate genes across the contigs and relative distance of the associated SNP marker and candidate genes were found using BARLEX database and alignment package of Lasergene core suit of DNASTAR program. The gene ontology (GO) terms of the putative candidate genes were assigned using the Uniprot database.

## Results

### Genotyping by sequencing and SNP detection

The genotyping by sequencing (GBS) pipeline resulted in a total of 2,028,787 sequence tags, of which 1,548,708 (76.3%) were aligned with unique positions across the barley chromosomes. The sequence reads aligned with unique positions were subjected to SNP calling across the genotypes, founding 67,508 (unfiltered) Hapmap SNPs. After applying the filtering criteria as described in material and methods, a total of 1370 polymorphic SNPs were retained and utilized in further analyses. These SNP were distributed across all seven barley chromosomes. The highest number of SNP (214) were found on chromosome 7H and the lowest on chromosome 4H (108). The details of these SNPs, their corresponding chromosomes and contigs information are summarized in Figure S1.

### Population structure

The population structure analysis grouped barley landraces into three subpopulations (Figures S2A–C). The membership coefficient assignment (≥90%) indicated that most of the individuals were grouped in the first two groups, whereas the third inferred cluster contained few individuals. The membership coefficient assignment also revealed that most of the landraces from different geographic regions were clustered in group 1 (30 accessions) and group 2 (63 accessions). However, all landraces that were assigned to group 3 (18) originated from Tigray, but one accession from this region was assigned to group two. Bale (89%), Arsi (83%), Sidamo (79%), Harerge (68%), and Welo (63%) were the regions that contained highly admixed individuals. In contrast, less than half of the accessions collected from Shewa, Tigray, Gonder, and Gojam contained less than 10% admixtures within each individual, which was derived from historical ancestors. This percentage value indicated that more than half of the barley individuals from these regions have a membership coefficient that assigned these accessions to a distinct group. After the membership coefficient was assigned to each individual, we also tested whether altitude classes (Class I: below 2000; Class II: 2001–2500; Class III: 2501–3000; Class IV: above 3000 m.a.s.l) were the basis for the detection of the three sub-populations. All but one of the barley accessions in group 3 and 80% of the accessions in group 1 were collected from altitude classes I and II; the rest (20%) were collected from altitude class III (Figure S2D). Unlike other groups, barley landraces in group 2 were collected from altitude class II (13%) and class III (68%), and all accessions collected from altitude class IV (19%).

**Figure 2:**
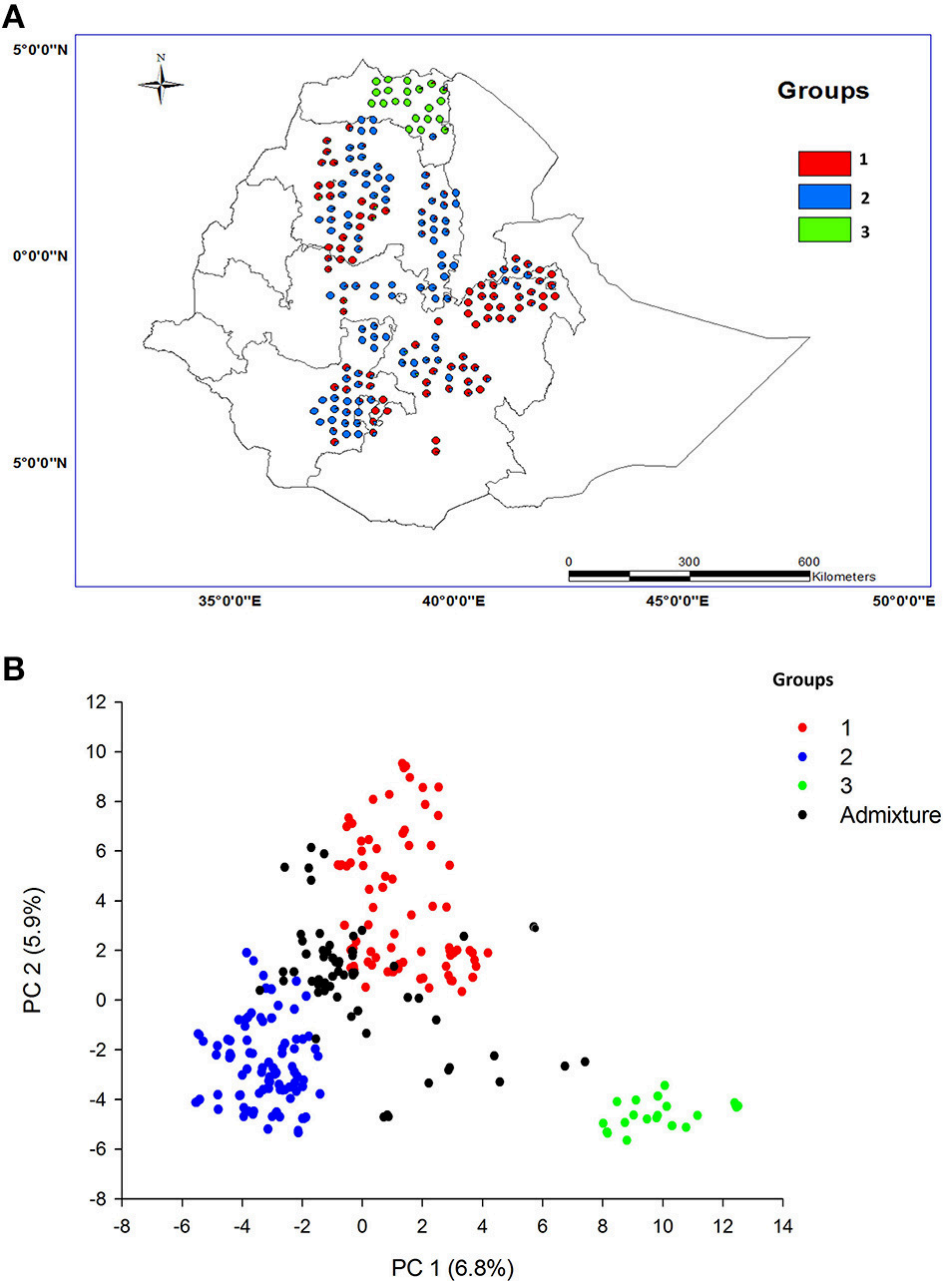
**Distribution of barley landraces and assignment of population membership coefficient along Ethiopian map (A)**. Each barley landrace was assigned to its respective inferred cluster based on the membership coefficient obtained from population structure analysis carried out using the model based software STRUCTURE. The scatter plot of principal components represented by the first and second principal components depicting the groupings of the barley individuals based on the subpopulations **(B)**.

To visualize the geographic distribution of the population structure, we plotted the pie chart of the membership coefficient on an Ethiopian map (Figure 2A). The distribution of the barley landraces based on their area of origin was associated with their groupings. Most of the landraces from the eastern part of Ethiopia (Harerge), Gojam, Sidamo, and Welo were clustered in group 1, whereas the landraces collected from the rest of the regions were assigned to group 2, except Tigray, which was assigned to group 3.

### Principal component analysis

Principal component analysis (PCA) reduced the variables into fewer components to explain most of the variation. Despite many eigenvalues, which were greater than one, we retained the first three principal components with variance of 15.03, 13.29, and 10.83. The proportions of variance explained by the respective principal components were 6.8, 5.9, and 4.9%. According to parallel analysis, the first three eigenvalues were sufficient for describing the grouping of the population. In order to visualize the pattern of the population grouping the first two principal components were plotted in 2-D. An assignment of individuals to their respective groups based on a ≥90% membership coefficient from population structure analysis resulted in approximately 57% of the individuals being categorized as admixtures (Figure 2B). Consequently, we assigned each barley individual to its respective group by considering its membership coefficient from the structure analysis and plotted the individuals based on the principal component values. In general, the first principal component separated groups one and three from group two, whereas the second principal component separated group one from the rest of the groups.

### Spatial population structure

**Figure 3:**
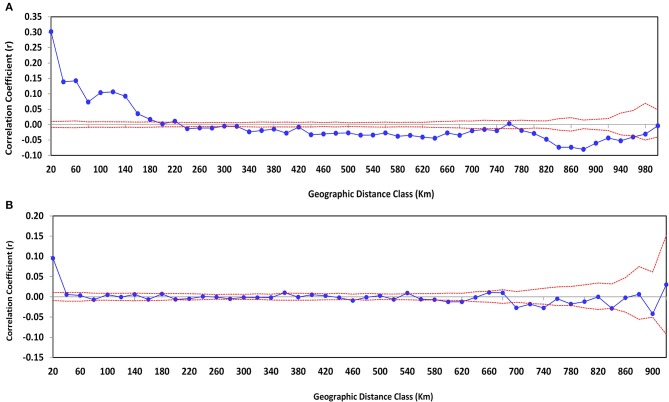
**Spatial autocorrelation correlogram plots**. The plot depicts results obtained from all geographic regions **(A)**, and after accessions collected from Tigray region were removed and isolation by distance was calculated for accessions from the rest of the regions **(B)**. The analysis considered geographic distances with even distance class of 20 km. Dashed lines encompass the 95% confidence interval of the null hypothesis, and each point represents the autocorrelation coefficient (r).

A spatial analysis was computed using the entire data and excluding the accessions collected from Tigray. First, the analysis was performed for the accessions from all regions, and it showed a significant spatial autocorrelation (Figure 3A). Further, this analysis revealed a significant and positive spatial autocorrelation for the closest accessions and a negative correlation for the accessions collected from a wide distance. The positive and weak correlation between genetic similarity and geographic distance in the first dataset was observed for the genotypes collected in a range of 180 km (*r* = 0.017, *p* = 0.001). The presence of negative correlation for accessions collected in a geographic distance range of 780 km (*r* = −0.013) to 960 km (*r* = −0.037) was observed. However, after the accessions collected from Tigray were removed, the positive correlation was detected at short distances ranging from 20 km (*r* = 0.095, *p* = 0.001) to 40 km (*r* = 0.006, *p* = 0.1) (Figure 3B). Although most of the distance classes showed no spatial autocorrelation, the overall result of the spatial analysis revealed the presence of weak spatial population structure at the shortest distances, thus indicating genetic similarity.

### Partitioning of genomic variation due to climate variables and geographic distance

**Figure 4:**
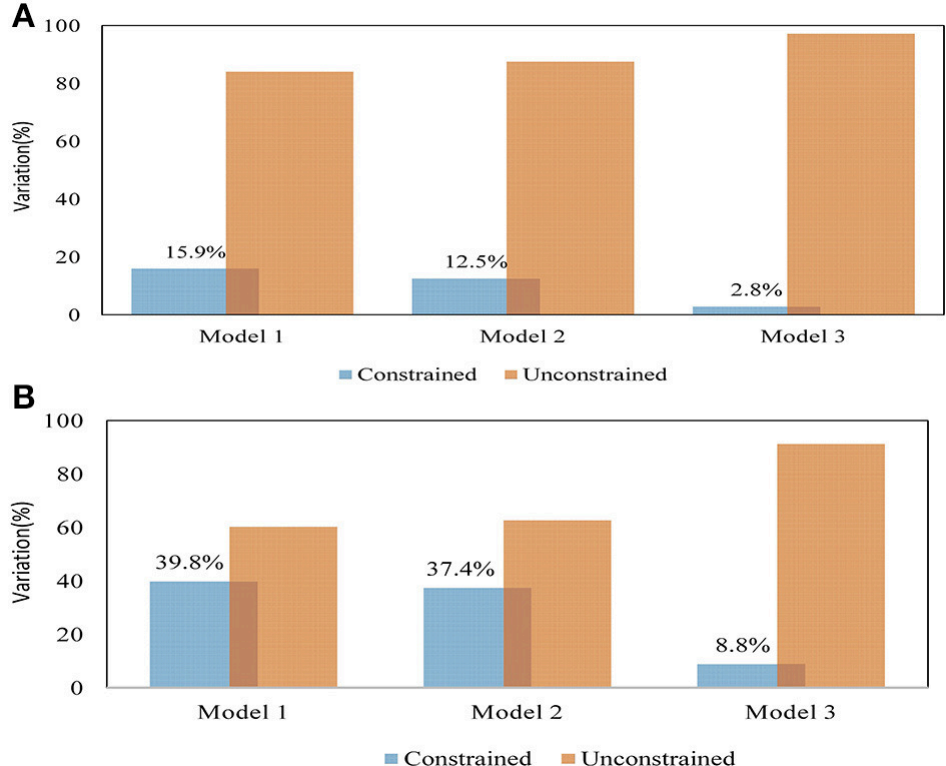
**The partial RDA variance partitioning was computed for entire dataset (A) and after accessions from Tigray region were excluded (B)**. The variance explained due to all variables (Model 1), the variance explained after controlling the effect introduced due to geographic distance (Model 2), and variance explained by geographic coordinates after the variance due to climate variables controlled (Model 3).

**Figure 5:**
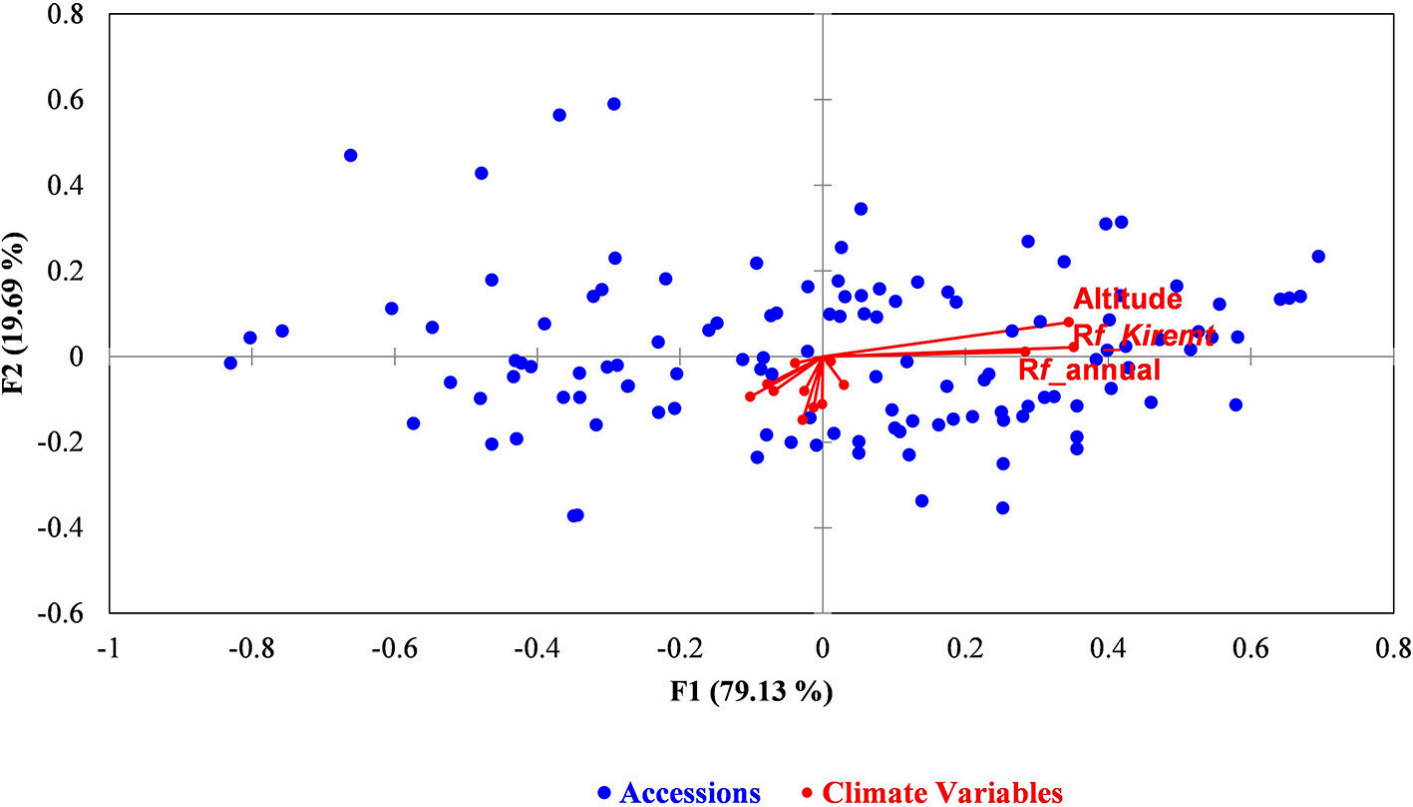
**Partial RDA analysis was performed to determine the relative contribution of climate and geographic variables shaping the genetic structure**. The biplot depicts the eigenvalues and lengths of eigenvectors for the RDA conditioned on geographic distance.

A partial redundancy analysis (RDA) was performed to partition the variations accounted by climatic and geographic variables. The RDA analysis for model 1, which used climate and geographic variables as explanatory variables, indicated that the variation due to climate and geographic variables (constrained) explained most of the variation compared with the residual variance (unconstrained) (Figure 4A). Partitioning of the total variance indicated that the climatic variables accounted for 40% of the explainable total variance after removing the effect due to geographic variables, whereas geographic variables explained 29% of the total variance after the effect of climatic variables was controlled. The combination of climate and geographic effects explained 61% of the total explainable variation. The variance partitioning indicated that in Model 1 (F1 = 38.4%, F2 = 34.64%, F3 = 26.71%) and Model 2 (F1 = 69.64%, F2 = 22.62%, F3 = 7.74%) the first three eigenvalues contributed 100% to the variation, in contrast to Model 3, where two of the eigenvalues contributed to the total explainable variation (F1 = 81.83%, F2 = 18.17%; Figures S3A–C). The RDA result obtained after excluding Tigray indicated the importance of the region in shaping the genetic diversity pattern of the entire population. Executing the RDA analysis without conditioning on any of the variables gave a close cumulative variance both with and without the Tigray region (60.6%; 57.2%) in the dataset (Figure 4B). A partial RDA analysis test for the full dataset yielded 40 and 29% for conditioning on geographic and climate variables, respectively. However, excluding Tigray from the dataset gave a value of 14.1 and 4.7% when conditioned on geographic and climate variables, respectively. The relative variances contributed by the presence of Tigray in the entire dataset conditioning on climate and geography were 35.3 and 16.5%, respectively. Furthermore, the eigenvalue results indicated low value and most of the variation was explained by residual variance (Figures S3D–F). We have also computed the partitioning among the climate variables while considering their major proportion in the total variance. It revealed that the variables altitude, Rf_*Kiremt* (rainfall in *Kiremt*) and Rf_annual explained most of the variation across the climatic variables (Figure 5, Table S3).

### Association analysis of climatic variables

The association analysis of SNP markers and climatic variables was performed using the Bayenv program. This analysis detected a total of 18 loci showing significant association with one or more climatic variables (Table 1). Among these, three loci were associated with variable altitude. Similar number of loci were associated with rainfall variables; Rf_*Bega* (1) and Rf*_Kiremt* (2). The highest number of loci were associated with minimum temperature variables; Mintemp_*Bega* (2), Mintemp_*Belg* (3), Mintemp_*Kiremt* (1), and Mintemp_aver (2) followed by maximum temperature variables; Maxtemp_*Bega* (2), Maxtemp_*Kiremt* (1), and Maxtemp_aver (1).

**Table 1:** **A summary of putative adaptive loci showing association with different climate variables identified using Bayenv analysis**.

**SNP ID**	**Chr**	**cM**	**BF**	**Rho (ρ)**	**Climatic variables**												
					**A**	**B**	**C**	**D**	**E**	**F**	**G**	**H**	**I**	**J**	**K**	**L**	**M**
Hv_SNP785	2H	65.59	3.05	0.52						^*^							
Hv_SNP4131	2H	123.94	3.26	0.60		^*^											
Hv_SNP8058	4H	77.48	8.22	0.37								^*^					
Hv_SNP51899	4H	78.61	3.97	0.44									^*^				
Hv_SNP51899	4H	78.61	4.21	0.44							^*^						
Hv_SNP594	4H	79.87	4.06	0.43									^*^				
Hv_SNP594	4H	79.87	3.00	0.49						^*^							
Hv_SNP594	4H	79.87	4.27	0.43							^*^						
Hv_SNP4616	5H	40.07	4.88	0.60													^*^
Hv_SNP4616	5H	40.07	5.51	0.51										^*^			
Hv_SNP15799	5H	129.65	5.00	0.45				^*^									
Hv_SNP56701	5H	169.38	3.68	0.51												^*^	
Hv_SNP31344	6H	100.42	3.05	0.46							^*^						
Hv_SNP30323	7H	55.74	4.55	0.47	^*^												
Hv_SNP23710	7H	109.92	5.44	0.54										^*^			
Hv_SNP23253	7H	124.58	3.14	0.45	^*^												
Hv_SNP58	U	U	3.20	0.44	^*^												
Hv_SNP32903	U	U	4.25	0.54				^*^									
		Total loci			3	1	–	2	–	2	3	1	2	2	–	1	1

*Where: A, Altitude; B, Rf_Bega; C, Rf _Belg; D, Rf_Kiremt; E, Rf_annual; F, Mintemp_Bega; G, Mintemp_Belg; H, Mintemp_Kiremt; I, Mintemp_aver; J, Maxtemp_Bega; K, Maxtemp_Belg; L, Maxtemp_Kiremt; M, Maxtemp_aver; U, Unknown*.

* *Indicates that the particular SNP showed correlation with that specific climate variable. BF (Bayes factor), Rho (ρ) (Spearman's rank correlation coefficient)*.

**Figure 6:**
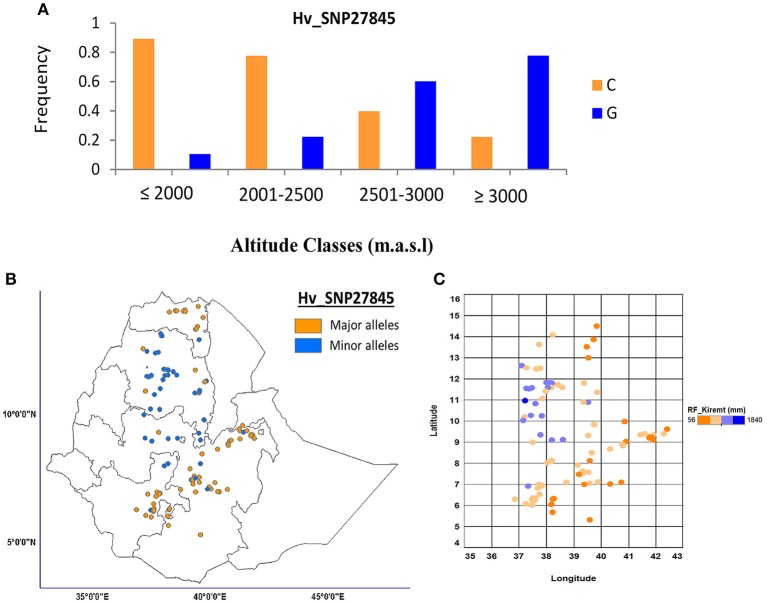
**Allele frequency of putative adaptive loci correlated with altitude classes and Rainfall in *Kiremt* as detected by LFMM**. The major and minor alleles of putative adaptive loci Hv_SNP27845 showed frequency pattern along the altitude classes **(A)**. The allele distribution depicting on the Ethiopian map **(B)** and the rainfall pattern along the coordinates of Ethiopia displayed in scatter plot **(C)**.

The association of SNP markers and climatic variables was also analyzed using a LFMM analysis. This analysis revealed the detection of 62 loci associated with the 13 selected climatic variables (Table 2). The highest number of loci (35) were associated with rainfall variables; Rf*_Bega* (10) and Rf*_Belg* (10), Rf*_Kiremt* (8) and Rf*_annual* (7). The second most number of loci were associated with variable altitude (9). In contrast, Mintemp_*Belg* and Mintemp_*Kiremt* were the only two climate variables that had one significant locus with *z* = 5.20 and *z* = 5.57, respectively. The highest number of common putative adaptive loci (6) were found for Rf_*Bega* and Rf_*Belg* followed by altitude and Rf_*Kiremt* (4). Among the loci commonly detected for altitude and Rf_*Kiremt*, we have selected the SNP locus Hv_SNP27845 with the highest significance level (*z* = 6.71). This locus was further illustrated to examine the allele frequency distribution along the altitude classes (Figure 6A) and rainfall as well as allele distribution over the country (Figure 6A). It showed that the most prevalent major allele at lowland was gradually decreased with an increase in the altitude and rainfall (Figures 6B,C). A complete summary of the LFMM analysis is presented in Table S4.

**Table 2:** **A summary of putative adaptive loci showing association with different climate variables identified using LFMM analysis**.

**SNP_ID**	**Chr**	**cM**	**Zscore**	**-log10 (*p*-value)**	**Climatic variables**												
					**A**	**B**	**C**	**D**	**E**	**F**	**G**	**H**	**I**	**J**	**K**	**L**	**M**
Hv_SNP57960	1H	7.22	4.26	4.68													^*^
Hv_SNP57963	1H	7.22	4.43	5.03										^*^			
**Hv_SNP9160**	1H	42.71	5.55	7.53		^*^	^*^		^*^								
Hv_SNP28572	1H	48.51	4.86	5.92	^*^			^*^									
**Hv_SNP28218**	1H	49.75	5.6	7.66		^*^	^*^										
Hv_SNP6094	1H	70.25	5.13	6.55				^*^									
Hv_SNP53255	1H	103.82	4.06	4.31			^*^										
Hv_SNP54198	1H	132.51	5.75	8.06		^*^											
Hv_SNP3374	2H	18.8	4.2	4.58		^*^											
**Hv_SNP27845**	2H	18.91	6.71	10.71	^*^			^*^									
Hv_SNP13837	2H	39.66	4.19	4.55										^*^			
**Hv_SNP4499**	2H	55.56	5.57	7.59	^*^			^*^		^*^	^*^	^*^	^*^				
Hv_SNP55036	2H	92.21	5.21	6.72					^*^								
**Hv_SNP4131**	2H	123.94	4.67	5.52										^*^		^*^	^*^
Hv_SNP25024	2H	138.6	4.32	4.8				^*^									
**Hv_SNP51311**	3H	83.59	4.37	4.91			^*^			^*^							
**Hv_SNP7771**	4H	18.48	5.89	8.41		^*^	^*^										
Hv_SNP54437	4H	19.9	4.17	4.52					^*^								
Hv_SNP15569	4H	35.13	5.96	8.6	^*^												
**Hv_SNP19635**	4H	60.55	4.48	5.12		^*^	^*^										
Hv_SNP25404	4H	91.18	4.18	4.53				^*^									
**Hv_SNP5505**	4H	105.49	4.68	5.55		^*^			^*^								
Hv_SNP34901	5H	13.77	4.68	5.54						^*^							
**Hv_SNP34783**	5H	77.08	6.27	9.44		^*^	^*^										
Hv_SNP37305	5H	79.13	4.23	4.64					^*^								
Hv_SNP30681	5H	80.35	4.06	4.31			^*^										
**Hv_SNP13299**	5H	95.9	5.01	6.27	^*^			^*^									
Hv_SNP27374	5H	161.08	4.42	5.00					^*^								
Hv_SNP64267	5H	164.72	5.88	8.39	^*^												
**Hv_SNP8419**	5H	164.72	4.57	5.31												^*^	^*^
**Hv_SNP36036**	5H	169.38	5.23	6.76		^*^	^*^			^*^							
**Hv_SNP65888**	5H	169.38	6.14	9.09			^*^			^*^			^*^				
Hv_SNP28364	6H	15.72	4.04	4.27											^*^		
Hv_SNP23365	6H	52.2	5.44	7.28		^*^											
Hv_SNP64219	6H	94.62	5.12	6.51	^*^												
Hv_SNP8527	7H	12.75	4.33	4.82				^*^									
Hv_SNP8936	7H	67.37	8.39	16.32	^*^												
Hv_SNP29190	7H	85.98	4.06	4.31					^*^								
Hv_SNP8273	7H	109.92	4.28	4.73	^*^												
			Total loci		9	10	10	8	7	5	1	1	2	3	1	2	3

*Where: A, Altitude; B, Rf_Bega; C, Rf _Belg; D, Rf_Kiremt; E, Rf_annual; F, Mintemp_Bega; G, Mintemp_Belg; H, Mintemp_Kiremt; I, Mintemp_aver; J, Maxtemp_Bega; K, Maxtemp_Belg; L, Maxtemp_Kiremt; M, Maxtemp_aver*.

* *Indicates that the particular SNP showed correlation with that specific climate variable. The underlined loci showed association with two climate variables whereas the underlined and bold loci are associated with three or more climate variables*.

### Outlier loci detection

**Figure 7:**
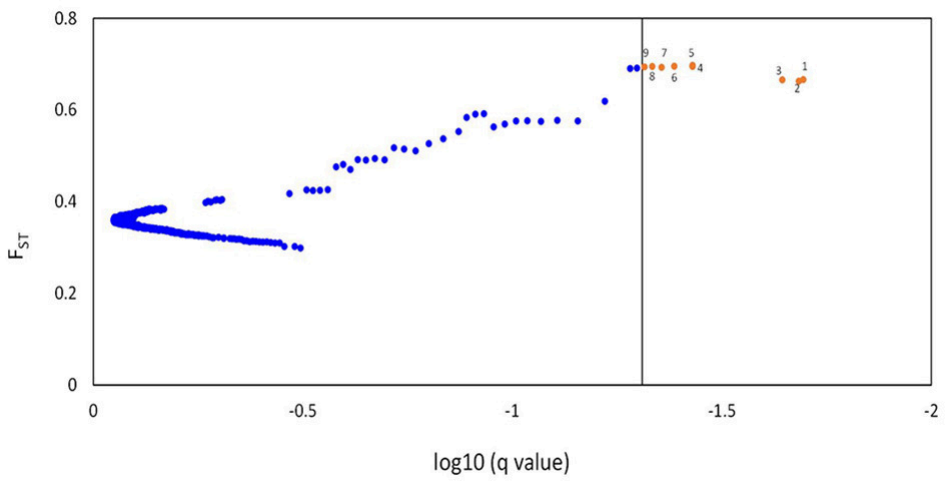
**A Bayesian based BayScan program were employed to scan for the presence of putative outlier loci affected by selection**. This plot presents *F*_ST_ against log 10(*q*-value), which is the FDR analog of the *p*-value. The line represents the threshold FDR = 0.05 and the red dots indicate the outlier loci which are potentially affected by directional selection.

The BayeScan method detected 12 and nine outlier loci (FDR = 0.05, prior 10:1) using a threshold of ≥70 and ≥90% ancestry coefficient of admixture for each barley individual, respectively (Figure S4, for the first approach). Of the nine loci detected using the second approach, six loci were also detected using the first approach. Three of the loci (Hv_SNP23336, Hv_SNP66136, and Hv_SNP27872) that were also detected with 100: one prior were considered for further analysis (Figure 7). The detected outlier loci showed a positive alpha value, which indicated directional selection. *F*_ST_-values ranged between 0.69 and 0.66 for Hv_SNP53122 and Hv_SNP23336, respectively. Notably, the three detected SNPs were mapped on the same position (70.68 cM) on chromosome 7H.

Altogether, none of the software shared common significant loci among them but one locus (Hv_SNP4131) was commonly detected between LFMM and Bayenv software (Figure S5).

### Detection of candidate genes

We have made an *in silico* analysis of the associated genomic regions to detect underlying putative candidate genes (Table 3). It revealed that all three SNP marker associated to altitude were found in single genomic contig (contig_46879) on chromosome 4H. These SNPs were found in the coding region (+1108 base pairs (bp) from ATG) of the sulfate transporter (*ST3.1*) gene. The SNP markers associated with altitude and Rf_*Kiremt* (rainfall in *Kiremt*) appear to underlie the L-lactate dehydrogenase (LDH) gene. These markers were around at +357 bp from ATG. The SNP locus (Hv_SNP4131) associated with maximum temperature (*Kiremt, Bega* and average) was found in the region of the cation\H+ exchanger (*CAX*) gene, +465 bp from ATG. Additionally, SNP loci on chromosome 2H associated with Maxtemp_*Bega*, were found next to each other in the putative promoter region (−2749 bp from ATG) of the universal stress responsive protein (*USP1*).

**Table 3:** **A summary of detected loci and identification of putative candidate genes**.

**Climate variables**	**Chr^a^**	**Associated SNP^b^**	**Genomic Contig^c^**	**Contig length^d^**	**Identity (%)^e^**	**Putative genes^f^**	**SNP to gene^g^**	**Accession Nr.^h^**	**Go term^i^**		
									**Biological function**	**Molecular function**	**Cellular components**
Altitude	4H	Hv_SNP11857	contig_46879	9106	96	Sulfhate transporter (*ST3.1*)	+1108*	AK358393	Sulfhate transport	Secondary active sulfate transmembrane transporter activity	Membrane
		Hv_SNP11859									
		Hv_SNP11860									
Altitude Rf_*Kiremt*	2H	Hv_SNP27843	contig_136338	8019	98	L-lactate dehydrogenase	+357*	AK375972	Response to stress	L-lactate dehydrogenase activity	Cytoplasm
		Hv_SNP27845									
						(*LDH*)					
Maxtemp_*Bega*	2H	Hv_SNP4131	contig_38530	6129	99	Cation\H^+^ exchanger (*CAX*)	+465*	AK373169	Cation transport	Cation transmembrane transporter activity	Membrane
Maxtemp_Kiremt											
Maxtemp_aver											
Maxtemp_*Bega*	2H	Hv_SNP13837	contig_49840	10470	99	Universal stress protein (*USP1*)	−2749	AY641412	Response to stress	Nucleotide binding	Cytosol
		Hv_SNP13839									

a Chr. Chromosome

b Associated SNPs loci

c Genomic contig containing associated SNPs and putative candidate genes

d Length of genomic contig in base pairs

e % similarity of SNP and genomic contig

f Putative candidate genes

g Position of SNP to putative genes in base pairs, + and − reveal the number of base pairs downstream and upstream of gene start site (ATG), associated SNP within the candidate genes are indicated as *

h Accession number NCBI

i *Gene ontology terms*.

## Discussion

### Population structure

The population structure analysis was computed using the STRUCTURE program and supported by the principal component analysis approach. The detected clusters did not completely reveal a geographically based population structure. Though accessions from 10 geographic regions were analyzed, the population structure analysis detected that three sub-populations contained different regions as one group. Hence, this result suggests the weak impact of geographic boundaries on the genetic structure of the barley population. A weak effect of political regions was reported for the morphological and genetic differences between major barley-growing areas of Ethiopia (Abebe et al., 2010; Abebe and Léon, 2013a). However, the pattern of clustering in the present study, was different compared with previous studies because of the difference in the number of barley genotypes, number of genetic markers and sampling strategy to genotype landraces. In the present study, we also replicated each landrace twice for genotyping to ensure high-quality genotyping data and to determine the genetic purity of the landraces that farmers have selected and established for barley cultivation. Among the inferred groups, the third cluster was aligned with one of the geographic regions. This region was Tigray, which is located in the northern part of Ethiopia and is frequently affected by drought because of a degraded environment and erratic rainfall (Abay et al., 2009). Farmers in this region have selected drought-resistant landraces to grow under water-limited conditions (Meze-Hausken, 2004). In addition, a decrease in rainfall northwards and eastwards from the high rainfall pocket area in the southwest has been reported (USDA, 2002). In the present study, Tigray was one of the regions having low percentage of admixed barley landraces (39%) and over 90% of the accessions from other regions were assigned to group 3. However, more than three quarters of the accessions from Arsi, Bale and Sidamo were considered admixed and were thus not assigned to a single cluster. These regions are known as the cereal belt of Ethiopia, which implies that a considerable amount of cereal production and marketing occurs in these areas. This leads to high genetic diversity in the region and gene flow between farmers' fields, resulting in admixed landraces (Negassa, 1985; Abebe et al., 2013b). The population structure coefficient sorted by altitude classes indicated that the accessions grouped in the first and second sub-populations originated in altitudes less than 2500 m.a.s.l. Except for a few accessions, the third sub-population contained accessions collected from the highlands (above 3000 m.a.s.l) of Ethiopia. In general, geographic regions and altitude classes were associated with different groups; however, the spatial distance was presumably not considered as the basis for the inferred clustering.

### Spatial genetic structure

Isolation by geographic distance occurs when the gene flow between organisms is restricted because of spatial isolation. The detection of a correlation between the genetic and geographic distance was described as isolation by distance (Wright, 1946). We also detected significant but weak isolation by distance for the dataset consisting of all the barley accessions and in the dataset where the accessions from the Tigray region were removed. The correlogram from the first dataset showed correlation with the geographic distance covering a wide range, whereas after excluding Tigray, a significant correlation was observed over a shorter distance. In this case, the accessions in a 40 km range were considered to be genetically similar and positively associated with geographic distance but the correlation was not different from zero. The population structure analysis grouped most of the accessions from this region in one group, indicating the presence of less shared ancestors among the accessions. Furthermore, the autocorrelation result revealed that the other regions are spatially isolated from Tigray because of its geographic location. Hence, the location of Tigray influenced the pattern of the spatial genetic structure in the studied population. The low percentage of admixture among the accessions was presumably associated with the low gene flow from the neighbor regions. This is attributed to the location, landscape, social and economic activity of the region. In general, the accessions from Tigray region affected the pattern of isolation by distance when all regions were considered for analysis. But the detected spatial correlation was weak and limited to a short distance to infer the presence of isolation by distance.

### Partitioning of genomic variation due to climate variables and geographic distance

The partial RDA was computed to estimate the proportion of variation explained by the environmental variables or by geographic distance alone or as the fraction of the variation shared by both variables. The variance partitioning for partial RDA models indicated that the variation contributed by climate variables were higher than the variation introduced due to geographic variables in both datasets (datasets are explained in material and methods). However, all the models showed significant association between the environmental variables and the genetic variation. The positive association of the climate variables with the genetic markers while controlling the variations due to geographic variables, thus suggests an important influence of climate diversity in shaping genetic variation (Temunovic et al., 2012). Similar findings were reported by Lasky et al. (2012) where they found a significant contribution of climate variables after controlling the spatial structure in *Arabidopsis thaliana*. They propose these variables as the selective gradients related to local adaptation across the species range. Unlike the climate variables the geographic coordinates showed low linear association with the genetic data indicating the influence of the spatial structure on the genetic variation of barley. Previously, Liu (1997) also found that climate factors accounted for 13% of the explained variation, whereas the geographic position was considered less important for algae colony thickness and colonization which are in agreement with the present study. Similar outcome was reported by McGaughran et al. (2014) who suggested the association between geography and genetic distance as an important determinant of genetic structure beyond genetic drift in isolated population. Moreover, comparing the results of both datasets revealed that accessions collected from Tigray region contributed more than a unit variance considering the contribution of the remaining regions to the environmental variation.

Further partitioning of variance explained due to the climate variables revealed altitude, total rainfall and rainfall of the main growing season as the main contributors of the detected genetic variation. Besides, the forward selection process retained altitude twice (in both datasets) as the first significant explanatory variable. This result indicated the importance of altitude in affecting the existing genetic variation in barley population. The importance of altitude in shaping and determining the climate variables and thus the genetic diversity in barley has been reported by Abebe et al. (2010) and Demissie and Bjornstad (1996). Similarly, Pyhäjärvi et al. (2013) controlled population structures using partial mantel analysis and found a significant effect of altitude in teosinte, the wild ancestor of maize. Besides, rainfall, which mostly depends on altitude, is one of the determinant factor in the genetic variation. Zhao et al. (2013) proposed annual rainfall as a major factor behind the genetic divergence and adaptation of Chinese wild rice (*Oryza rufipogon)*. Hence, the variance partitioning of the significant climate variables emphasized the importance of altitude in shaping the ecological diversity and evolutionary aspect of different plants.

### Climatic adaptations

Natural selection plays a major role in shaping the available genetic variation of a population and thereby determines local adaptation (Kawecki and Ebert, 2004). It also changes the allele frequency when individuals with the same fitness trait survive and increase in number. In this study, we observed a similar situation in allele distribution of the detected putative adaptive loci in response to different climate variables. The association of climate variables with SNP markers using Bayenv and LFMM returned several significant loci in relation to all climate variables, indicating that the variables were the important climate factors that affect selection pressure. Most of the loci detected using LFMM software were associated with rainfall variables followed by altitude, indicating the importance of these variables in determining local adaptation. In Bayenv analysis most loci were correlated with temperature variables followed by altitude and rainfall. Partial RDA analysis also indicated that altitude, Rf_*Kiremt* and Rf_annual were the most important climate variables; most of the variation originated from these variables. De Kort et al. (2014) reported strong associations between outlier loci and temperature using LFMM in the tree species *Alnus glutinosa*. LFMM detected a locus (Hv_SNP27845) showing correlation with altitude and rainfall variables which explained most of the variation in partial RDA. The pattern of decreasing frequency of the major alleles as a function of increasing altitude presumes the presence of directional selection, which leads to local adaptation. The minor alleles were observed in highland areas with high rainfall, indicating the importance of altitude in determining other climate factors. The prevalence of the major allele among the genotypes that were collected below 2500 m.a.s.l. in low rainfall areas was presumably due to local selection (Figure 6A). In this case, because of directional selection, the advantageous alleles increased in frequency relative to others and eventually became fixed (Bose and Bartholomew, 2013). Altitude affects phenology, the distribution and type of disease and the prevalence of frost in different crops of Ethiopia. In the highlands, barley matures quite late, and it takes as long as seven to 8 months to mature (Tanto and Demissie, 2001), whereas in the lowlands and in the *Belg* season, barley matures early, within 3–4 months (Mulatu and Grando, 2011). The special adaptation of barley to highlands makes the crop the most valuable cereal for the survival of the farmers living in the highlands as it is the only crop cultivated across those regions (Lakew et al., 1997). The highlands of Ethiopia are described as sunny during the day and cold at night with occurrences of frost, particularly during the *Bega* season (USDA, 2002). In general, altitude plays a major role in the determination of morphological novelties of different crops in Ethiopia (Engels, 1994; Abebe et al., 2010), and it affects the ecological variables and, thus, local adaptation. *Kiremt* (main rainy season) rains occur during June-September, accounting for 50–80% of the annual rainfall over the Ethiopian regions. The most severe droughts are usually related to a failure of the *Kiremt* rainfall to meet Ethiopia's agricultural water demands (Korecha and Barnston, 2007). In general, directional selection occurs when natural selection favors a single phenotype, and the allele frequency thus shifts in one direction. The loci that were identified as adaptive loci presumably underlie the phenotypic variation that affect fitness in different environments (Nunes et al., 2011).

### Detection of candidate genes

Although genes and phenotypes are in a causal relationship, dissecting the genetic components of a phenotype is not simple. Through the advent of genome-wide DNA markers and sequenced genomes, it has become feasible to uncover this relationship precisely and dissect the hidden genetic regulations in the expression of a phenotype at the gene level. In the present study, we utilize genome-wide SNP markers to dissect those footprints associated with barley adaptation to landscape and climatic variables. The associated SNP markers loci were then searched in the database for the putative genes. To this end, we are proposing four putative candidate genes due to their tight linkage with the associated SNP markers as well as due to likelihood of their functional linkage with a given climatic variable. For instance, the significant loci associated with altitude and rainfall variables underlie putative sulfate and L-lactate dehydrogenase genes. A number of studies suggested the role of sulfate genes in nutrient transport for plant growth as well as for environmental adaptation like drought and salinity stress (Hawkesford and Buchner, 2001; Gallardo et al., 2014). In Arabidopsis, lactate dehydrogenase genes are involved in adaptation to hypoxic stress (reduced oxygen because of water logging or higher altitudes) by switching plants from aerobic respiration to anaerobic fermentation (Dolferus et al., 2008). These results, seems to be in line with the present study where we found a putative association of L-lactate dehydrogenase gene with altitude and rainfall. Similarly, cation\H+ exchanger (*CAX*) and universal stress protein (*HvUSP1*) appeared as candidates for adaptation to higher temperature. Plants trigger the expression of a specialized protein called the heat shock or stress protein against climatic conditions such as higher temperature (Vierling, 1991; Parsell and Lindquist, 1993; Gupta et al., 2010). These proteins are then involved in the maintenance of cell membrane stability, capturing the reactive oxygen species (ROS), synthesis of antioxidants, accumulation and osmoregulation of osmoticum (Wahid et al., 2007). We believe that these data reveal a primary insight into the identification of primary evolutionary candidate genes mediating adaptation to important landscape and climatic variables across Ethiopia. However, further experiments are needed to confirm the precise role of these candidate genes in the process of local adaptation in barley.

Taken together, the present study has successfully analyzed the association between genetic markers and environmental factors to determine their effect on the explainable genetic variation. We identified climate and geographic variables as important explanatory aspects of genetic variation followed by altitude and rainfall as underlying cause of climatic variation. Hence, the detected correlation between environmental variables and genetic markers can help to understand the phenomenon of natural selection, yet, conducting the common garden experiment to verify the result will provide the strong evidence for the underlying phenotypic traits. In general, this study has successfully demonstrated how landscape genomics contribute to uncover the genetic components (genes) and evolutionary processes affecting adaptation. In conclusion, we assume that the detected candidate loci were associated with local adaptation that showed selective responses to important climatic variables.

### Conflict of interest statement

The authors declare that the research was conducted in the absence of any commercial or financial relationships that could be construed as a potential conflict of interest.
