# Supplementary material for: Landscape genomics reveal signatures of local adaptation in barley (Hordeum vulgare L.)
Source: Front Plant Sci. 2015 Oct 2;6:813. doi: 10.3389/fpls.2015.00813 (PMC4591487; doi:10.3389/fpls.2015.00813)
Supplement: Supplementary file 1 [file DataSheet1.DOCX]

**Supporting Information**

**Table S1** List of all accessions, regions and coordinates which was used for the study.

| **Accession Number** | **Adminstrative regions** | **Altitude (m)** | **Latitude** | **Longitude** |
| --- | --- | --- | --- | --- |
| 213538 | Arsi | 2980 | 07° 27' N | 39° 15' E |
| 216989 | Arsi | 2600 | 07° 29' N | 39° 11' E |
| 229990 | Arsi | 3120 | 07° 38' N | 39° 19' E |
| 217112 | Arsi | 2920 | 07° 29' N | 39° 15' E |
| 217008 | Arsi | 2690 | 08° 07' N | 39° 35' E |
| 223959 | Arsi | 3150 | 07° 33' N | 39° 22' E |
| 229992 | Arsi | 2400 | 07° 35' N | 39° 32' E |
| 213707 | Arsi | 2640 | 07° 06' N | 38° 44' E |
| 218944 | Arsi | 2260 | 07° 55' N | 39° 08' E |
| 237006 | Arsi | 2810 | 07° 19' N | 39° 16' E |
| 237002 | Arsi | 2350 | 07° 19' N | 39° 16' E |
| 232217 | Arsi | 2220 | 08° 30' N | 39° 39' E |
| 217065 | Bale | 2650 | 07° 23' N | 39° 32' E |
| 217082 | Bale | 2470 | 07° 06' N | 40° 44' E |
| 243187 | Bale | 2150 | 07° 00' N | 40° 23' E |
| 243185 | Bale | 1990 | 07° 01' N | 40° 23' E |
| 243178 | Bale | 2200 | 07° 01' N | 40° 19' E |
| 230591 | Bale | 2400 | 07° 00' N | 39° 23' E |
| 217081 | Bale | 2450 | 07° 07' N | 40° 02' E |
| 230640 | Bale | 2240 | 07° 12' N | 39° 57' E |
| 212826 | Bale | 2390 | 07° 22' N | 40° 12' E |
| 230653 | Bale | 2610 | 07° 07' N | 39° 53' E |
| 212967 | Gamo Gofa | 2350 | 06° 51' N | 37° 18' E |
| 241685 | Gamo Gofa | 2160 | 06° 01' N | 37° 29' E |
| 233038 | Gamo Gofa | 2380 | 06° 05' N | 37° 15' E |
| 241684 | Gamo Gofa | 2200 | 06° 21' N | 37° 30' E |
| 233028 | Gamo Gofa | 2050 | 05° 55' N | 37° 20' E |
| 217054 | Gamo Gofa | 2700 | 06° 21' N | 37° 36' E |
| 216990 | Gamo Gofa | 2960 | 06° 16' N | 37° 35' E |
| 216999 | Gamo Gofa | 3030 | 06° 17' N | 37° 35' E |
| 217004 | Gamo Gofa | 2830 | 06° 32' N | 37° 45' E |
| 204807 | Gamo Gofa | 2590 | 06° 14' N | 37° 34' E |
| 217051 | Gamo Gofa | 2900 | 06° 17' N | 37° 28' E |
| 233023 | Gamo Gofa | 2800 | 06° 15' N | 37° 33' E |
| 216992 | Gamo Gofa | 2960 | 06° 16' N | 37° 35' E |
| 219770 | Gojam | 2450 | 11° 33' N | 37° 16' E |
| 225997 | Gojam | 2000 | 11° 32' N | 37° 22' E |
| 216978 | Gojam | 2345 | 11° 04' N | 37° 51' E |
| 225266 | Gojam | 2260 | 10° 16' N | 37° 26' E |
| 225265 | Gojam | 2260 | 10° 16' N | 37° 26' E |
| 217059 | Gojam | 2550 | 10° 15' N | 37° 50' E |
| 216009 | Gojam | 2260 | 10° 16' N | 37° 26' E |
| 216975 | Gojam | 3090 | 10° 50' N | 37° 36' E |
| 216957 | Gojam | 2880 | 10° 58' N | 37° 13' E |
| 213708 | Gojam | 2780 | 10° 58' N | 37° 13' E |
| 238378 | Gojam | 2530 | 10° 02' N | 37° 09' E |
| 204788 | Gonder | 2350 | 11° 36' N | 38° 34' E |
| 225994 | Gonder | 2010 | 12° 32' N | 37° 16' E |
| 235883 | Gonder | 2900 | 12° 30' N | 37° 46' E |
| 225991 | Gonder | 2385 | 11° 25' N | 37° 58' E |
| 217010 | Gonder | 2090 | 12° 38' N | 37° 06' E |
| 235880 | Gonder | 2280 | 12° 28' N | 37° 38' E |
| 243308 | Gonder | 2358 | 11° 35' N | 38° 03' E |
| 216959 | Gonder | 2730 | 11° 50' N | 38° 00' E |
| 219744 | Gonder | 2400 | 11° 35' N | 37° 28' E |
| 235888 | Gonder | 2800 | 13° 20' N | 37° 38' E |
| 235885 | Gonder | 2260 | 13° 38' N | 37° 44' E |
| 235886 | Gonder | 2940 | 13° 19' N | 37° 38' E |
| 216966 | Gonder | 2810 | 11° 49' N | 38° 08' E |
| 243284 | Gonder | 2990 | 11° 48' N | 38° 12' E |
| 217019 | Gonder | 3000 | 11° 44' N | 38° 25' E |
| 225986 | Gonder | 2890 | 11° 36' N | 38° 11' E |
| 216778 | Harerge | 2330 | 09° 37' N | 42° 24' E |
| 232351 | Harerge | 2380 | 09° 37' N | 42° 24' E |
| 241121 | Harerge | 2080 | 09° 59' N | 40° 51' E |
| 223301 | Harerge | 2030 | 08° 41' N | 40° 19' E |
| 230506 | Harerge | 2530 | 09° 29' N | 42° 15' E |
| 241678 | Harerge | 2350 | 08° 54' N | 40° 46' E |
| 208675 | Harerge | 2600 | 09° 14' N | 41° 50' E |
| 219101 | Harerge | 2090 | 09° 02' N | 40° 54' E |
| 216787 | Harerge | 2510 | 09° 15' N | 41° 46' E |
| 241682 | Harerge | 2440 | 09° 06' N | 41° 54' E |
| 241683 | Harerge | 2220 | 09° 06' N | 41° 54' E |
| 216810 | Harerge | 2430 | 09° 21' N | 41° 26' E |
| 241680 | Harerge | 2340 | 08° 54' N | 40° 46' E |
| 241679 | Harerge | 2350 | 08° 54' N | 40° 46' E |
| 204787 | Harerge | 2200 | 09° 24' N | 41° 35' E |
| 241120 | Harerge | 2200 | 09° 02' N | 40° 54' E |
| 208676 | Harerge | 2420 | 09° 12' N | 41° 45' E |
| 230499 | Harerge | 2250 | 09° 24' N | 42° 17' E |
| 241677 | Harerge | 1990 | 08° 50' N | 40° 47' E |
| 241681 | Harerge | 2230 | 06° 21' N | 41° 54' E |
| 241676 | Harerge | 1990 | 08° 50' N | 40° 47' E |
| 208904 | Shewa | 2450 | 09° 00' N | 37° 30' E |
| 208911 | Shewa | 2900 | 09° 12' N | 37° 12' E |
| 216934 | Shewa | 2900 | 09° 06' N | 38° 12' E |
| 216923 | Shewa | 2730 | 09° 07' N | 38° 36' E |
| 224954 | Shewa | 2830 | 09° 50' N | 39° 45' E |
| 235528 | Shewa | 3250 | 08° 07' N | 38° 11' E |
| 235550 | Shewa | 2910 | 08° 02' N | 38° 02' E |
| 235531 | Shewa | 3200 | 08° 07' N | 38° 12' E |
| 213522 | Shewa | 2600 | 07° 21' N | 37° 47' E |
| 224912 | Shewa | 3280 | 09° 19' N | 39° 31' E |
| 236801 | Shewa | 3200 | 09° 51' N | 39° 44' E |
| 212507 | Shewa | 3130 | 10° 03' N | 39° 35' E |
| 219321 | Sidamo | 2520 | 05° 19' N | 39° 35' E |
| 219305 | Sidamo | 2220 | 06° 03' N | 38° 11' E |
| 219306 | Sidamo | 2940 | 05° 41' N | 38° 13' E |
| 225179 | Sidamo | 2100 | 06° 57' N | 37° 51' E |
| 219304 | Sidamo | 1930 | 06° 20' N | 38° 16' E |
| 217107 | Sidamo | 1880 | 06° 50' N | 37° 41' E |
| 219330 | Sidamo | 1950 | 07° 00' N | 37° 44' E |
| 225184 | Sidamo | 2100 | 06° 57' N | 37° 51' E |
| 225181 | Sidamo | 2100 | 06° 57' N | 37° 51' E |
| 217101 | Sidamo | 2100 | 06° 55' N | 37° 49' E |
| 233053 | Sidamo | 1850 | 06° 18' N | 38° 14' E |
| 233052 | Sidamo | 1850 | 06° 18' N | 38° 14' E |
| 234344 | Tigray | 2200 | 14° 08' N | 38° 33' E |
| 219915 | Tigray | 2600 | 14° 10' N | 38° 55' E |
| 238357 | Tigray | 2920 | 14° 30' N | 39° 50' E |
| 219935 | Tigray | 1940 | 14° 05' N | 38° 14' E |
| 235298 | Tigray | 2000 | 13° 24' N | 39° 23' E |
| 235293 | Tigray | 2060 | 13° 27' N | 39° 26' E |
| 234341 | Tigray | 2100 | 14° 07' N | 38° 51' E |
| 234343 | Tigray | 2180 | 14° 08' N | 38° 30' E |
| 234347 | Tigray | 2100 | 14° 08' N | 38° 34' E |
| 221712 | Tigray | 1990 | 13° 31' N | 39° 28' E |
| 235294 | Tigray | 2060 | 13° 26' N | 39° 25' E |
| 238355 | Tigray | 2910 | 14° 30' N | 39° 50' E |
| 242092 | Tigray | 2950 | 13° 00' N | 39° 32' E |
| 238373 | Tigray | 2130 | 13° 52' N | 39° 43' E |
| 238353 | Tigray | 1970 | 13° 04' N | 38° 04' E |
| 215212 | Welo | 2950 | 10° 54' N | 39° 31' E |
| 204805 | Welo | 2350 | 11° 20' N | 39° 46' E |
| 215699 | Welo | 2240 | 11° 22' N | 39° 51' E |
| 204819 | Welo | 2650 | 10° 54' N | 39° 21' E |
| 215695 | Welo | 2540 | 11° 20' N | 39° 47' E |
| 224921 | Welo | 2920 | 10° 57' N | 39° 33' E |
| 215210 | Welo | 2900 | 11° 00' N | 39° 33' E |
| 224948 | Welo | 2860 | 11°48' N | 39° 22' E |

**Table S2** Summary of barley landraces according to geographic regions and altitude classes across Ethiopia. Each barley landrace was used in two replications.

| Altitude classes (meters above sea level) | | | | | |
| --- | --- | --- | --- | --- | --- |
| Regions | Class I  (below 2 000) | Class II  (2 001-2 500) | Class III  (2 501-3 000) | Class IV  (above 3 000) | Total |
| Arsi | - | 8 | 12 | 4 | 24 |
| Bale | 2 | 14 | 4 | - | 20 |
| Gojam | 2 | 10 | 8 | 2 | 22 |
| Gamo Gofa | - | 9 | 14 | 2 | 25 |
| Gonder | - | 13 | 19 | - | 32 |
| Harerge | 4 | 30 | 8 | 2 | 44 |
| Shewa | - | 2 | 12 | 8 | 22 |
| Sidamo | 12 | 11 | 1 | - | 24 |
| Tigray | 8 | 15 | 8 | - | 31 |
| Welo | - | 6 | 10 | - | 16 |
| Total | 28 | 118 | 96 | 18 | 260 |

**Table S3** The eigenvectors of partial RDA conditioned on geographic distance to control the spatial effect. The first two axis and the contribution towards the total variation is presented blow.

| Climate variables | F1 | F2 |
| --- | --- | --- |
| Altitude | 0.34 | 0.08 |
| Rf_*Bega* | -0.04 | -0.02 |
| Rf_*Belg* | 0.01 | -0.01 |
| Rf_*Kiremt* | 0.35 | 0.02 |
| Rf_annual | 0.28 | 0.01 |
| Mintemp_*Bega* | -0.03 | -0.15 |
| Mintemp_*Belg* | -0.01 | -0.12 |
| Mintemp_*Kiremt* | 0.03 | -0.07 |
| Mintemp_aver | 0.00 | -0.11 |
| Maxtemp_*Bega* | -0.03 | -0.08 |
| Maxtemp_*Belg* | -0.08 | -0.07 |
| Maxtemp_*Kiremt* | -0.10 | -0.09 |
| Maxtemp_aver | -0.07 | -0.08 |

**Table S4** List of loci detected using LFMM showing association with climate varibles along with SNP codes, the chromosomal postion and the contigs with the Z-score and –log10 (p-value).

| Climate variables | SNP_ID | Chr | cM | Contigs | Zscore | -log10(p-value) |
| --- | --- | --- | --- | --- | --- | --- |
| Altitude |  |  |  |  |  |  |
|  | Hv_SNP28572^a^ | 1H | 48.51 | morex_contig_137643 | 4.86 | 5.92 |
|  | Hv_SNP29016 | 1H | 48.80 | morex_contig_138669 | 5.47 | 7.34 |
|  | Hv_SNP29018 | 1H | 48.80 | morex_contig_138669 | 5.59 | 7.65 |
|  | Hv_SNP29019 | 1H | 48.80 | morex_contig_138669 | 5.59 | 7.64 |
|  | Hv_SNP27843^b^ | 2H | 18.91 | morex_contig_136338 | 6.12 | 9.03 |
|  | Hv_SNP27845^c^ | 2H | 18.91 | morex_contig_136338 | 5.94 | 8.54 |
|  | Hv_SNP4499^d^ | 2H | 55.56 | morex_contig_38888 | 4.47 | 5.11 |
|  | Hv_SNP15569 | 4H | 35.13 | morex_contig_52709 | 5.96 | 8.60 |
|  | Hv_SNP11857 | 4H | 73.40 | morex_contig_46879 | 5.34 | 7.03 |
|  | Hv_SNP11859 | 4H | 73.40 | morex_contig_46879 | 4.96 | 6.16 |
|  | Hv_SNP11860 | 4H | 73.40 | morex_contig_46879 | 4.90 | 6.02 |
|  | Hv_SNP13299^e^ | 5H | 95.90 | morex_contig_48912 | 4.84 | 5.89 |
|  | Hv_SNP64267 | 5H | 164.72 | morex_contig_2548020 | 5.88 | 8.39 |
|  | Hv_SNP64268 | 5H | 164.72 | morex_contig_2548020 | 5.20 | 6.70 |
|  | Hv_SNP64219 | 6H | 94.62 | morex_contig_2547875 | 5.12 | 6.51 |
|  | Hv_SNP8935 | 7H | 67.37 | morex_contig_43261 | 8.39 | 16.30 |
|  | Hv_SNP8936 | 7H | 67.37 | morex_contig_43261 | 8.39 | 16.32 |
|  | Hv_SNP23579 | 7H | 70.68 | morex_contig_88696 | 5.52 | 7.46 |
|  | Hv_SNP4484 | 7H | 70.68 | morex_contig_38874 | 5.56 | 7.57 |
|  | Hv_SNP30234 | 7H | 70.68 | morex_contig_146571 | 5.76 | 8.07 |
|  | Hv_SNP35703 | 7H | 70.68 | morex_contig_244067 | 6.62 | 10.43 |
|  | Hv_SNP8272 | 7H | 109.92 | morex_contig_42648 | 4.16 | 4.50 |
|  | Hv_SNP8273 | 7H | 109.92 | morex_contig_42648 | 4.28 | 4.73 |
| Rf_*Bega* |  |  |  |  |  |  |
|  | Hv_SNP9160^o^ | 1H | 42.71 | morex_contig_43545 | 4.57 | 5.31 |
|  | Hv_SNP28218^p^ | 1H | 49.75 | morex_contig_136896 | 4.95 | 6.14 |
|  | Hv_SNP28220^q^ | 1H | 49.75 | morex_contig_136896 | 4.78 | 5.76 |
|  | Hv_SNP54198 | 1H | 132.51 | morex_contig_1569224 | 5.75 | 8.06 |
|  | Hv_SNP3371 | 2H | 18.80 | morex_contig_37667 | 4.19 | 4.56 |
|  | Hv_SNP3374 | 2H | 18.80 | morex_contig_37667 | 4.20 | 4.58 |
|  | Hv_SNP7771^r^ | 4H | 18.48 | morex_contig_42154 | 4.49 | 5.14 |
|  | Hv_SNP19635^s^ | 4H | 60.55 | morex_contig_62174 | 4.48 | 5.12 |
|  | Hv_SNP5505^t^ | 4H | 105.49 | morex_contig_39705 | 4.30 | 4.77 |
|  | Hv_SNP56853 | 5H | 62.50 | morex_contig_1583288 | 4.38 | 4.93 |
|  | Hv_SNP34783^u^ | 5H | 77.08 | morex_contig_223763 | 6.27 | 9.44 |
|  | Hv_SNP57004 | 5H | 80.57 | morex_contig_1585042 | 4.82 | 5.83 |
|  | Hv_SNP21016 | 5H | 92.99 | morex_contig_66384 | 5.20 | 6.70 |
|  | Hv_SNP21017 | 5H | 92.99 | morex_contig_66384 | 5.27 | 6.87 |
|  | Hv_SNP41351 | 5H | 93.63 | morex_contig_369324 | 4.39 | 4.95 |
|  | Hv_SNP36036^l^ | 5H | 169.38 | morex_contig_245134 | 4.96 | 6.16 |
|  | Hv_SNP40115^m^ | 5H | 169.38 | morex_contig_339707 | 4.76 | 5.72 |
|  | Hv_SNP1003^n^ | 5H | 169.38 | morex_contig_6900 | 4.72 | 5.63 |
|  | Hv_SNP1004^h^ | 5H | 169.38 | morex_contig_6900 | 5.02 | 6.28 |
|  | Hv_SNP23365 | 6H | 52.20 | morex_contig_86195 | 5.44 | 7.28 |
| Rf_*Belg* |  |  |  |  |  |  |
|  | Hv_SNP9160^o^ | 1H | 42.71 | morex_contig_43545 | 4.56 | 5.28 |
|  | Hv_SNP28218^p^ | 1H | 49.75 | morex_contig_136896 | 5.60 | 7.66 |
|  | Hv_SNP28220^q^ | 1H | 49.75 | morex_contig_136896 | 5.51 | 7.46 |
|  | Hv_SNP30881 | 1H | 95.61 | morex_contig_158271 | 4.01 | 4.21 |
|  | Hv_SNP53255 | 1H | 103.82 | morex_contig_1566033 | 4.06 | 4.31 |
|  | Hv_SNP1057 | 1H | 103.82 | morex_contig_6962 | 4.05 | 4.29 |
|  | Hv_SNP51311^j^ | 3H | 83.59 | morex_contig_1560072 | 4.37 | 4.91 |
|  | Hv_SNP51312^k^ | 3H | 83.59 | morex_contig_1560072 | 4.02 | 4.23 |
|  | Hv_SNP7771^r^ | 4H | 18.48 | morex_contig_42154 | 5.89 | 8.41 |
|  | Hv_SNP19635^s^ | 4H | 60.55 | morex_contig_62174 | 4.21 | 4.60 |
|  | Hv_SNP31198 | 5H | 62.50 | morex_contig_158756 | 5.36 | 7.08 |
|  | Hv_SNP7701 | 5H | 62.50 | morex_contig_42055 | 5.23 | 6.78 |
|  | Hv_SNP8095 | 5H | 75.90 | morex_contig_42476 | 4.29 | 4.74 |
|  | Hv_SNP34783^u^ | 5H | 77.08 | morex_contig_223763 | 4.67 | 5.52 |
|  | Hv_SNP30678 | 5H | 80.35 | morex_contig_157345 | 4.05 | 4.29 |
|  | Hv_SNP30679 | 5H | 80.35 | morex_contig_157345 | 4.03 | 4.25 |
|  | Hv_SNP30681 | 5H | 80.35 | morex_contig_157345 | 4.06 | 4.31 |
|  | Hv_SNP36036^l^ | 5H | 169.38 | morex_contig_245134 | 4.40 | 4.97 |
|  | Hv_SNP65888^i^ | 5H | 169.38 | morex_contig_2553377 | 4.47 | 5.10 |
| Rf_*Kiremt* |  |  |  |  |  |  |
|  | Hv_SNP28572^a^ | 1H | 48.51 | morex_contig_137643 | 4.35 | 4.87 |
|  | Hv_SNP6093 | 1H | 70.25 | morex_contig_40245 | 5.10 | 6.46 |
|  | Hv_SNP6094 | 1H | 70.25 | morex_contig_40245 | 5.13 | 6.55 |
|  | Hv_SNP27843^b^ | 2H | 18.91 | morex_contig_136338 | 6.57 | 10.29 |
|  | Hv_SNP27845^C^ | 2H | 18.91 | morex_contig_136338 | 6.71 | 10.71 |
|  | Hv_SNP4499^d^ | 2H | 55.56 | morex_contig_38888 | 4.94 | 6.11 |
|  | Hv_SNP25024 | 2H | 138.60 | morex_contig_106745 | 4.32 | 4.80 |
|  | Hv_SNP25404 | 4H | 91.18 | morex_contig_113413 | 4.18 | 4.53 |
|  | Hv_SNP13299^e^ | 5H | 95.90 | morex_contig_48912 | 5.01 | 6.27 |
|  | Hv_SNP21403 | 7H | 0.39 | morex_contig_67562 | 4.05 | 4.28 |
|  | Hv_SNP7075 | 7H | 3.82 | morex_contig_41382 | 4.11 | 4.41 |
|  | Hv_SNP8527 | 7H | 12.75 | morex_contig_42889 | 4.33 | 4.82 |
| Rf_annual |  |  |  |  |  |  |
|  | Hv_SNP9160^o^ | 1H | 42.71 | morex_contig_43545 | 5.55 | 7.53 |
|  | Hv_SNP16498 | 1H | 46.60 | morex_contig_54415 | 4.61 | 5.39 |
|  | Hv_SNP6610 | 1H | 46.81 | morex_contig_40818 | 4.02 | 4.24 |
|  | Hv_SNP55036 | 2H | 92.21 | morex_contig_1571783 | 5.21 | 6.72 |
|  | Hv_SNP54437 | 4H | 19.90 | morex_contig_1570147 | 4.17 | 4.52 |
|  | Hv_SNP54440 | 4H | 19.90 | morex_contig_1570147 | 4.16 | 4.50 |
|  | Hv_SNP5505^t^ | 4H | 105.49 | morex_contig_39705 | 4.68 | 5.55 |
|  | Hv_SNP64272 | 4H | 112.33 | morex_contig_2548052 | 5.29 | 6.92 |
|  | Hv_SNP50967 | 4H | 113.14 | morex_contig_1559316 | 4.01 | 4.22 |
|  | Hv_SNP37305 | 5H | 79.13 | morex_contig_274373 | 4.23 | 4.64 |
|  | Hv_SNP27374 | 5H | 161.08 | morex_contig_135662 | 4.42 | 5.00 |
|  | Hv_SNP29190 | 7H | 85.98 | morex_contig_139013 | 4.06 | 4.31 |
|  | Hv_SNP29192 | 7H | 85.98 | morex_contig_139013 | 4.05 | 4.29 |
|  | Hv_SNP29194 | 7H | 85.98 | morex_contig_139013 | 4.04 | 4.28 |
| Mintemp_*Bega* |  |  |  |  |  |  |
|  | Hv_SNP4499^d^ | 2H | 55.56 | morex_contig_38888 | 4.37 | 4.90 |
|  | Hv_SNP51311^j^ | 3H | 83.59 | morex_contig_1560072 | 4.16 | 4.49 |
|  | Hv_SNP51312^k^ | 3H | 83.59 | morex_contig_1560072 | 4.36 | 4.89 |
|  | Hv_SNP34901 | 5H | 13.77 | morex_contig_228317 | 4.68 | 5.54 |
|  | Hv_SNP34903 | 5H | 13.77 | morex_contig_228317 | 4.37 | 4.91 |
|  | Hv_SNP35488 | 5H | 14.25 | morex_contig_241513 | 4.49 | 5.14 |
|  | Hv_SNP35489 | 5H | 14.25 | morex_contig_241513 | 4.48 | 5.14 |
|  | Hv_SNP36036^l^ | 5H | 169.38 | morex_contig_245134 | 5.23 | 6.76 |
|  | Hv_SNP40115^m^ | 5H | 169.38 | morex_contig_339707 | 5.04 | 6.33 |
|  | Hv_SNP1003^n^ | 5H | 169.38 | morex_contig_6900 | 5.16 | 6.60 |
|  | Hv_SNP1004^h^ | 5H | 169.38 | morex_contig_6900 | 5.54 | 7.51 |
|  | Hv_SNP65888^i^ | 5H | 169.38 | morex_contig_2553377 | 6.14 | 9.09 |
| Mintemp_*Belg* |  |  |  |  |  |  |
|  | Hv_SNP4499^d^ | 2H | 55.56 | morex_contig_38888 | 5.20 | 6.70 |
| Mintemp_*Kiremt* |  |  |  |  |  |  |
|  | Hv_SNP4499^d^ | 2H | 55.56 | morex_contig_38888 | 5.57 | 7.59 |
| Mintemp_aver |  |  |  |  |  |  |
|  | Hv_SNP4499^d^ | 2H | 55.56 | morex_contig_38888 | 5.27 | 6.86 |
|  | Hv_SNP1004^h^ | 5H | 169.38 | morex_contig_6900 | 4.18 | 4.54 |
|  | Hv_SNP65888^i^ | 5H | 169.38 | morex_contig_2553377 | 4.99 | 6.21 |
| Maxtemp_*Bega* |  |  |  |  |  |  |
|  | Hv_SNP57963 | 1H | 7.22 | morex_contig_1599009 | 4.43 | 5.03 |
|  | Hv_SNP13837 | 2H | 39.66 | morex_contig_49840 | 4.19 | 4.55 |
|  | Hv_SNP13839 | 2H | 39.66 | morex_contig_49840 | 4.16 | 4.50 |
|  | Hv_SNP4131^f^ | 2H | 123.94 | morex_contig_38530 | 4.05 | 4.29 |
| Maxtemp_*Belg* |  |  |  |  |  |  |
|  | Hv_SNP28360 | 6H | 15.72 | morex_contig_137060 | 4.03 | 4.25 |
|  | Hv_SNP28364 | 6H | 15.72 | morex_contig_137060 | 4.04 | 4.27 |
| Maxtemp_*Kiremt* |  |  |  |  |  |  |
|  | Hv_SNP4131^f^ | 2H | 123.94 | morex_contig_38530 | 4.67 | 5.52 |
|  | Hv_SNP8419^g^ | 5H | 164.72 | morex_contig_42773 | 4.50 | 5.17 |
| Maxtemp_aver |  |  |  |  |  |  |
|  | Hv_SNP57960 | 1H | 7.22 | morex_contig_1599009 | 4.26 | 4.68 |
|  | Hv_SNP4131^f^ | 2H | 123.94 | morex_contig_38530 | 4.59 | 5.35 |
|  | Hv_SNP8419^g^ | 5H | 164.72 | morex_contig_42773 | 4.57 | 5.31 |

The SNP IDs followed by the small letters indicated that the locus is detected more than once. The same letter was given for a SNP marker which shows association with different climate variables.

**Figure S1** A histogram presenting total number of SNPs and contigs across chromosomes 1H to 7H and with unknown position.

**
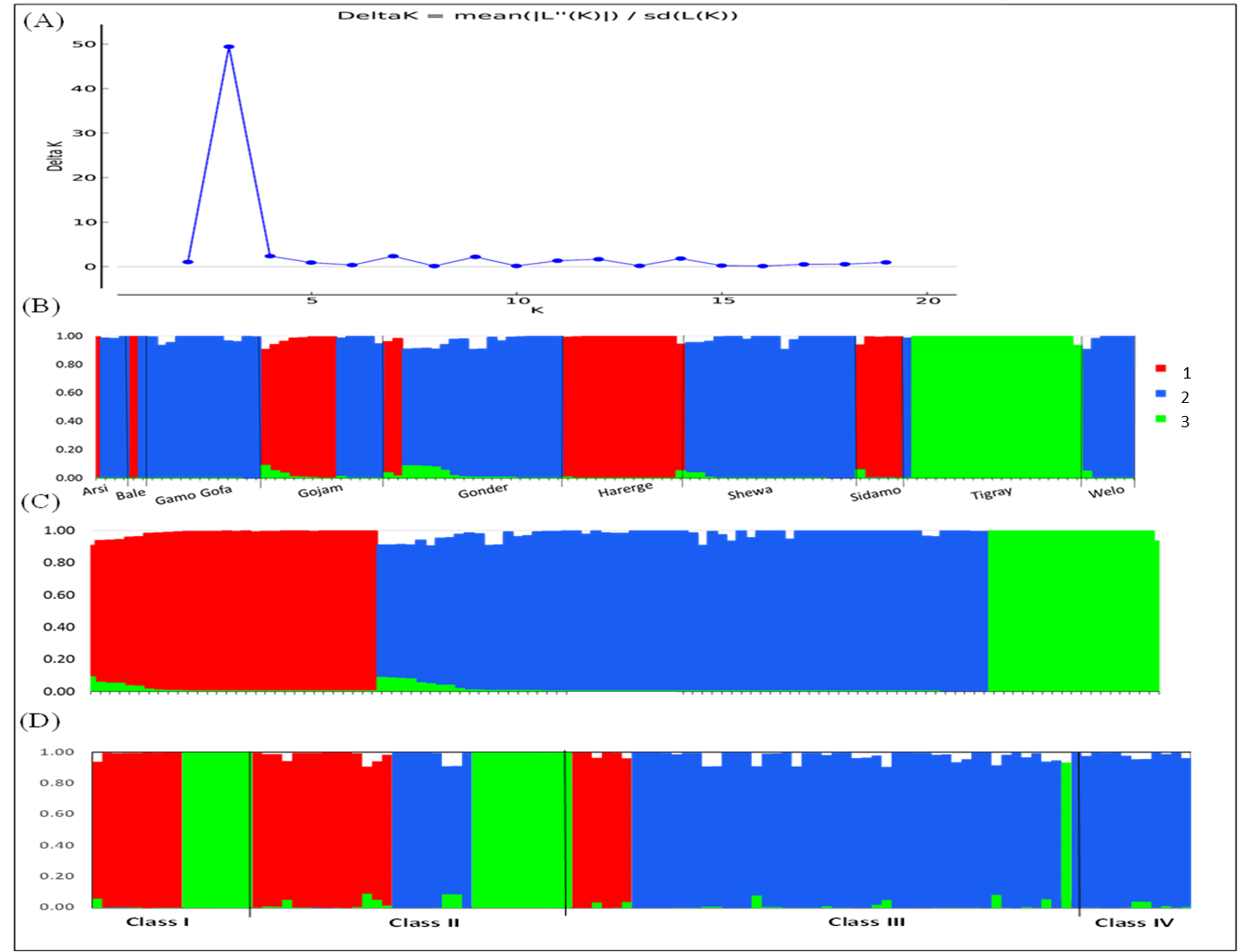
**

**Figure S2** Bayesian based population structure analysis of barley landraces computation was resulted in three groups (A). The accessions were sorted according to their region of origin, and the vertical lines represent barley individuals broken into K = 3 the area covered by the colours within a bar indicating the membership coefficient (B), and again accessions sorted based on the K values are presented (C). The population structure membership sorted based on altitude Class I (below 2,000 m.a.s.l), Class II (2,001-2,500 m.a.s.l), Class III (2,501-3,000 m.a.s.l) and Class IV (above 3,000 m.a.s.l) (D).

**
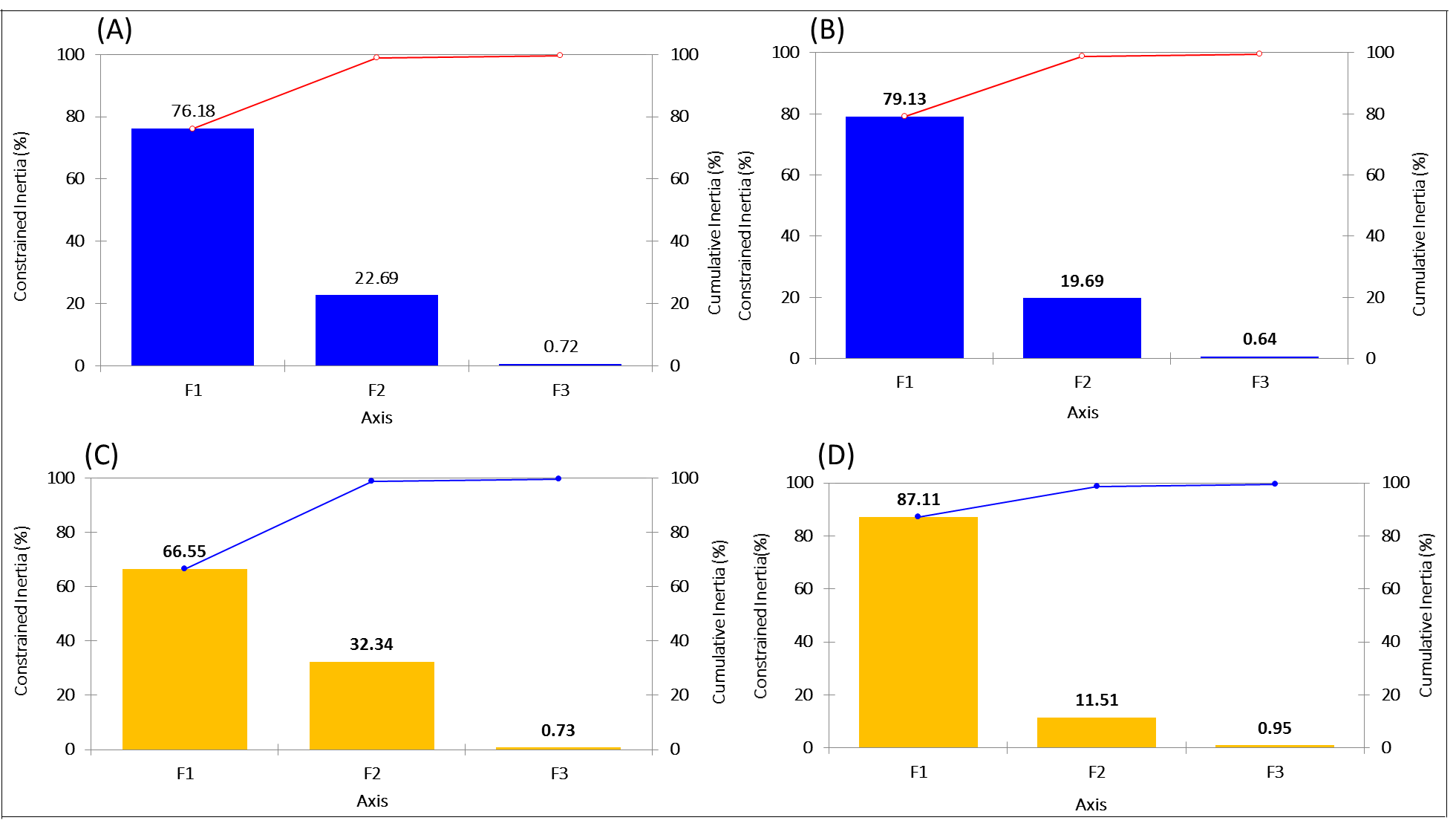
**

**Figure S3** Partitioning of the constrained variance from global and partial RDA analysis. The first three RDA axes which contributed to a total variance for the first (A and B) and the second dataset (D and E). Figures A and D indicated the variance accounted due to both climate variables and geographic coordinates together. Whereas, figures B and C shows the total explainable genetic variance due to climate variables after the variance accounted by geographic coordinates removed.

**Figure S4** A Bayesian based Bayescan program used to scan for the presence of selection. This plot presents F_ST_ against log 10 (q-value), which is the FDR analogue of the p-value. The line represents the threshold FDR = 0.05 and the red dots indicated the outlier loci which are affected by directional selection.


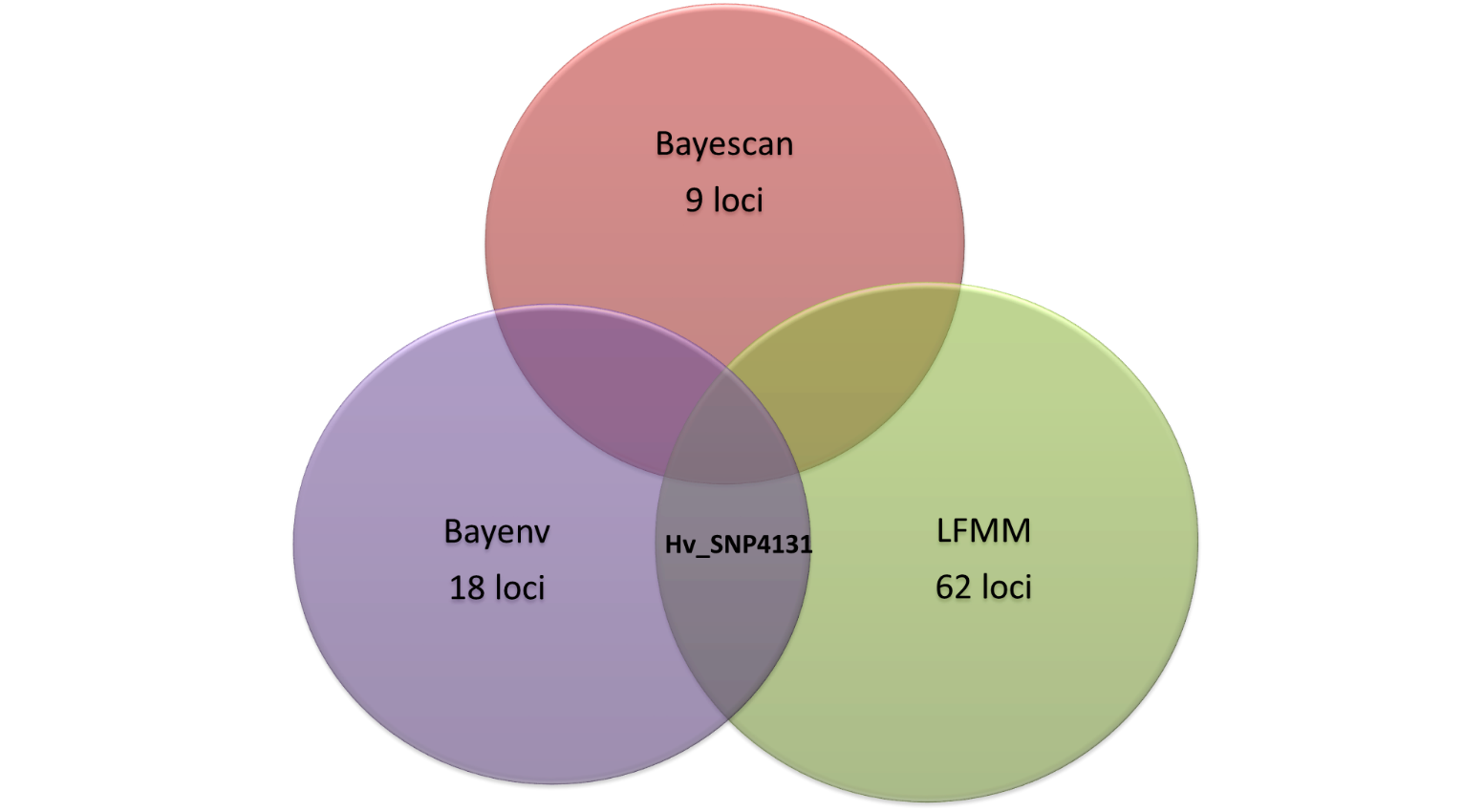


**Figure S5** Venn diagram showing the numbers of putative loci detected by respective software. The marker locus Hv_SNP4131 was detected by Bayenv and LFMM software.
